# Immortalized stem cell-derived hepatocyte-like cells: An alternative model for studying dengue pathogenesis and therapy

**DOI:** 10.1371/journal.pntd.0008835

**Published:** 2020-11-20

**Authors:** Kessiri Kongmanas, Nuntaya Punyadee, Kasima Wasuworawong, Adisak Songjaeng, Tanapan Prommool, Yongyut Pewkliang, Siriphan Manocheewa, Somchai Thiemmeca, Khanit Sa-ngiamsuntorn, Chunya Puttikhunt, Kym Francis Faull, Suradej Hongeng, Panisadee Avirutnan

**Affiliations:** 1 Division of Dengue Hemorrhagic Fever Research, Department of Research and Development, Faculty of Medicine Siriraj Hospital, Mahidol University, Bangkok, Thailand; 2 Siriraj Center of Research Excellence in Dengue and Emerging Pathogens, Faculty of Medicine Siriraj Hospital, Mahidol University, Bangkok, Thailand; 3 Siriraj Metabolomics and Phenomics Center, Faculty of Medicine Siriraj Hospital, Mahidol University, Bangkok, Thailand; 4 Molecular Biology of Dengue and Flaviviruses Research Team, Medical Molecular Biotechnology Research Group, National Center for Genetic Engineering and Biotechnology, National Science and Technology Development Agency, Bangkok, Thailand; 5 Excellent Center for Drug Discovery, Faculty of Science, Mahidol University, Bangkok, Thailand; 6 Graduate Program in Immunology, Department of Immunology, Faculty of Medicine Siriraj Hospital, Mahidol University, Bangkok, Thailand; 7 Department of Biochemistry, Faculty of Pharmacy, Mahidol University, Bangkok, Thailand; 8 Pasarow Mass Spectrometry Laboratory, Jane and Terry Semel Institute for Neuroscience and Human Behavior, David Geffen School of Medicine, University of California Los Angeles, California, United States of America; 9 Department of Pediatrics, Faculty of Medicine Ramathibodi Hospital, Mahidol University, Bangkok, Thailand; University of Hawaii at Manoa, UNITED STATES

## Abstract

Suitable cell models are essential to advance our understanding of the pathogenesis of liver diseases and the development of therapeutic strategies. Primary human hepatocytes (PHHs), the most ideal hepatic model, are commercially available, but they are expensive and vary from lot-to-lot which confounds their utility. We have recently developed an immortalized hepatocyte-like cell line (imHC) from human mesenchymal stem cells, and tested it for use as a substitute model for hepatotropic infectious diseases. With a special interest in liver pathogenesis of viral infection, herein we determined the suitability of imHC as a host cell target for dengue virus (DENV) and as a model for anti-viral drug testing. We characterized the kinetics of DENV production, cellular responses to DENV infection (apoptosis, cytokine production and lipid droplet metabolism), and examined anti-viral drug effects in imHC cells with comparisons to the commonly used hepatoma cell lines (HepG2 and Huh-7) and PHHs. Our results showed that imHC cells had higher efficiencies in DENV replication and NS1 secretion as compared to HepG2 and Huh-7 cells. The kinetics of DENV infection in imHC cells showed a slower rate of apoptosis than the hepatoma cell lines and a certain similarity of cytokine profiles to PHHs. In imHC, DENV-induced alterations in levels of lipid droplets and triacylglycerols, a major component of lipid droplets, were more apparent than in hepatoma cell lines, suggesting active lipid metabolism in imHC. Significantly, responses to drugs with DENV inhibitory effects were greater in imHC cells than in HepG2 and Huh-7 cells. In conclusion, our findings suggest superior suitability of imHC as a new hepatocyte model for studying mechanisms underlying viral pathogenesis, liver diseases and drug effects.

## Author summary

A model system resembling normal human liver cells is needed for advancement of hepatotropic infectious disease research. Here we show that immortalized cells (imHC) derived from human stem cells have a higher efficiency of DENV replication and a lower rate of cell death in response to DENV infection than the cancer cell-derived model systems currently used. The imHC also have active fat metabolism and respond well to anti-viral drug treatment, making them an attractive model for the initial stage of drug discovery and testing.

## Introduction

Understanding the pathogenesis of liver diseases is fundamental to successful development of effective therapy. An appropriate hepatocyte model is essential for doing this because it facilitates research into the molecular basis of the disease. The most ideal liver cell model for such studies is primary human hepatocytes (PHHs), but the acquisition of normal human hepatocytes is problematic because of biological and ethical considerations. Moreover, the PHHs can only be cultured for a short period of time, restricting their utility (or suitability) for most studies. Human hepatoma cell lines, such as HepG2 and Huh-7 are readily available, easily maintained and have been commonly used as hepatic models [1–3]. However, such carcinoma-derived cell lines have genetic and characteristic limitations, including the lack of drug-metabolizing enzymes and unusually active rates of cellular division [4]. It is necessary to take these limitations into consideration when designing experiments and interpreting results when using these hepatoma cell lines.

Recently, non-cancerous hepatocyte-like cells derived from different stem cell sources have been introduced as an alternative hepatic model that can be applied to studies of drug metabolism, hepatotoxicity and even viral infections [5,6]. However, after induction of differentiation, the life span and differentiated phenotypes of these hepatocyte-like cells are still generally limited [7]. To overcome this restriction, our research group generated “immortalized stem cell-derived hepatocyte-like cells (imHC)” through transduction of the precursor human mesenchymal stem cells with lentiviruses carrying human telomerase reverse transcriptase (hTERT) and polycomb complex protein (Bmi-1) genes [8,9]. The imHC maintain hepatic phenotypes and metabolic functions throughout their life span (> 6 months) [8,9]. Based on the non-cancerous origin and long-term viability of imHC, we propose that imHC could serve as a good substitute for PHHs to model liver diseases and test for drug efficiency and/or hepatotoxicity.

Liver is a target of infections by a number of viruses, including flaviviruses such as yellow fever virus [10] and dengue virus (DENV) [11]. DENV has 4 serotypes (DENV-1, DENV-2, DENV-3, and DENV-4), and each serotype contains a single positive stranded RNA encoding 3 structural proteins (capsid, pre-membrane, envelope) and 7 non-structural proteins (NS1, NS2A, NS2B, NS3, NS4A, NS4B, and NS5) [12]. Approximately 360 million people worldwide are infected by DENV each year, and about 96 milllion of the infected cases manifest clinically [13]. The clinical manifestations of DENV infection are classified into mild dengue fever (DF), and a severe form known as dengue hemorrhagic fever (DHF) or dengue shock syndrome (DSS) [14]. Severe DHF/DSS is life-threatening and is usually accompanied by liver injury, thrombocytopenia and vascular leakage. Deposition of complement proteins and expression of DENV antigens and genomes are observed in hepatocytes and Kupffer cells of fatal dengue cases, suggesting that hepatocytes are one of the major cell types in the liver responsible for viral replication and immune responses during DENV infection [15].

Herein we investigated the potential use of imHC as an in vitro hepatic model for studying DENV infection. We characterized and compared susceptibility to DENV infection, selected protein and lipid profiles as well as cellular responses of imHC upon DENV infection and drug treatment to the commonly used hepatoma cell lines and PHHs.

## Methods

### Ethics statement

The written informed consent was obtained from healthy donors and the blood collection protocol was approved by the ethics committee, Faculty of Medicine, Siriraj Hospital, Mahidol University, Thailand [Siriraj-IRB COA no. Si707/2016, Protocol number: 632/2559 (EC2)].

### Cells and viruses

imHC, derived from human mesenchymal stem cells and immortalized by transduction of hTERT plus Bmi-1 as previously described [9], were grown in Dulbecco's Modified Eagles Medium/Nutrient Mixture F-12 (DMEM/F-12, Gibco) supplemented with 10% fetal bovine serum (FBS, Gibco). Human hepatocellular carcinoma cell lines, Huh-7 and HepG2 were obtained from the American Type Culture Collection (ATCC). HepG2 was grown in Dulbecco's Modified Eagles Medium (DMEM) supplemented with 10% FBS with 1% non-essential amino acids (Gibco) and 1 mM sodium pyruvate (Biochrome). Huh-7 was grown in DMEM supplemented with 10% FBS and 1% non-essential amino acids. African green monkey kidney epithelial cell line (Vero) was grown in Minimum Essential Media (MEM, Gibco) supplemented with 10% FBS. Human glioma cell line (U87-MG) was obtained from ATCC and maintained in DMEM supplemented with 10% FBS. *Aedes albopictus* C6/36 mosquito cells were grown in Leibovitz-15 medium (L-15, Gibco) supplemented with 10% FBS and 10% tryptose phosphate broth (Sigma). All culture media for cell lines were supplemented with 2 mM L-glutamine (Sigma), 100 U/ml penicillin G, and 100 μg/ml streptomycin sulfate (Sigma). PHHs (Lot numbers Hu8272 and Hu1604) obtained from Thermofisher Scientific Inc. were plated in collagen-I coated plates and cultured in William’s E medium with supplements, according to the company’s instructions. Peripheral blood mononuclear cells were isolated from heparinized blood of healthy volunteers by density gradient centrifugation on Lymphoprep.

The viruses used in this study include laboratory and clinical strains of four DENV serotypes. The laboratory strains were the Hawaii, 16681, H87 and H241 of DENV-1, DENV-2, DENV-3 and DENV-4, respectively. For clinical isolates, DENV-1 was isolated in-house from autopsy liver tissue of a fatal DSS case admitted to Siriraj Hospital; DENV-2, DENV-3 and DENV-4, which were isolated from the plasma of patients admitted to Queen Sirikit National Institute of Child Health between 2000 and 2002 [16], were provided as gifts from the Armed Forces Research Institute of Medical Sciences (AFRIMS). To generate DENV virus stock, the viruses were propagated in C6/36 cells. The viral titers were determined by the focus forming unit (FFU) assay on Vero cells as previously described [17].

### Determination of DENV-2 binding efficiency and effects of heparin on the viral binding of hepatic cell lines

The three hepatic cell types (imHC, Huh-7 and HepG2) were cultured in 24-well plates in their complete media as described above. The cells were detached from the wells with 8 mM ethylenediaminetetraacetic acid (EDTA) and 10% FBS in PBS (EDTA solution). The cells (5 × 10^5^) were incubated with different amounts of DENV-2 at multiplicity of infection (MOI) of 1, 5 and 10 or without DENV (as a negative control) for 1 hour (h) at 4°C, and washed once with washing buffer [DMEM (for HepG2 and Huh-7) or DMEM/F12 (for imHC) with 2% FBS]. DENV-2 bound onto the cell surface was detected by immunofluorescent staining with anti-envelope (E) protein monoclonal antibody (mAb) clone 4G2 (1 h, 4°C) and goat anti-mouse IgG conjugated with Alexa Fluor 488 [30 minutes (min), 4°C]. After washing, the cells were resuspended in washing buffer containing 0.2 mg/ml of propidium iodide (PI, BD Biosciences). The levels of DENV bound to the viable cells (negative PI staining) were analyzed by flow cytometry using a LSRFortessa flow cytometer (BD Biosciences). The data were further analyzed using Flowjo software.

Since heparan sulfate has previously been shown to be a major DENV receptor on human liver cells [18], its role for initial DENV binding of imHC was further investigated using heparin (a structural analog of heparan sulfate) as a competitive inhibitor for DENV binding [18,19]. The three hepatic cell types were incubated with DENV-2 at MOI of 5 for 1 h at 4°C in the presence of heparin at different concentrations (0, 0.1, 1, 5, 10 and 20 μg/ml), and the bound DENV levels were determined as described above.

### Detection of DENV receptors

Cells with known DENV receptor expression were used as positive controls for this experiment [i.e., HEK-293T cells stably expressing T-cell immunoglobulin and mucin domain (TIM)-1 and TIM-4, which were kindly provided by Dr. Ali Amara, the AP-HP Saint-Louis Hospital Research Institute, Paris, France; U87-MG for TYRO3; Vero for AXL]. The control and three hepatic cell lines were cultured in 24-well plates and detached from the wells by the EDTA solution. The cells (5 × 10^5^) were washed with washing buffer and incubated with 10% donkey serum (D9663, Sigma) in PBS for 30 min at 4°C to block non-specific binding. The cells were washed once and incubated (1 h at 4°C) with goat polyclonal antibodies specific for TIM-1 (AF1750), TIM-4 (AF2929), AXL (AF154), and TYRO3/Dtk (AF859) (R&D Systems). Normal polyclonal goat IgG (AB-108-C, R&D Systems) was also used in place of the primary antibodies to serve as the negative staining control. The cells were washed once and incubated with donkey anti-goat IgG conjugated with Alexa Fluor 488 (30 min at 4°C). The cells were then resuspended in washing buffer containing 0.2 mg/ml PI and analyzed by flow cytometry.

Western blotting analysis was also performed to determine expression of DENV receptors (i.e., TIM-1, TIM-4, Tyro3, AXL and Syndecan-1) in PHHs, three hepatic cell lines and the positive control cells. Antibodies specific to TIM-1, TIM-4 and AXL were the same as those described for flow cytometry. Mouse mAb against human Syndecan-1 (sc-12765) was obtained from Santa Cruz Biotechnology Inc.

### Kinetics of DENV antigen and virion production in hepatocytes

The efficiencies of DENV replication/production in three different cell types (imHC, Huh-7 and HepG2) were compared by kinetic experiments. The three cell lines were plated in 24-well plates (2 x 10^5^ cells/well) and cultured in their corresponding media overnight prior to mock infection or DENV-2 infection with different MOIs (0.1, 1 and 5). At 24 and 48 h post-infection, the culture supernatants were collected and the cells were washed five times with plain media. Measurement of DENV replication levels in the cells was based on intracellular NS3 expression as described below. Infectious virion levels in the supernatants were quantified by FFU assay as previously described [17], and secreted NS1 levels were measured by ELISA as described below.

For determination of cell associated virus levels, the infected cell monolayers were treated with acidic glycine solution (pH 3.0) for 1 min at room temperature (RT) to inactivate extracellular viruses, as previously described [20] and were then extensively washed with their corresponding plain media. Medium supplemented with 20% FBS (200 μl) was added to the acid-washed cells, followed by three cycles of freezing at −70°C and thawing at 4°C. After the cellular debris was removed by centrifugation at 10,000 x g for 10 min, cell associated virus levels in the cell lysates were measured by FFU assay.

### DENV antigen detection

The cells were detached from the wells by incubation in 0.1% trypsin and 2.5 mM EDTA in PBS (3 min, 37°C) and subjected to DENV antigen detection. Briefly, for immunofluorescent staining the cells were fixed with 3.7% formaldehyde in PBS, permeabilized with 0.1% Triton X-100/PBS (Sigma), and incubated for 1 h at RT with 20 μg/ml of antibodies specific to DENV antigens: anti-NS3 mAb clone E1D8 (a gift from Dr. Eva Harris, University of California, Berkeley, USA), anti-E mAb clone 4G2, anti-NS1 mAb clone 2G6. After washing, the cells were incubated with Alexa Fluor 647-conjugated anti-mouse IgG for 30 min at RT. The numbers of cells staining positive for DENV antigens were assessed by flow cytometry.

Western blotting analysis was also performed to detect NS3, NS1, E, and capsid (C) proteins for the kinetics and drug treatment experiments. Rabbit polyclonal antibody to NS3 (GTX124252) was obtained from GeneTex Inc. Mouse mAbs specific to E (clone 4G2), NS1 (clone 1F11) and C (clone 1C4) were produced in our laboratory.

### Western blotting analysis

The cell lysates were prepared in RIPA buffer containing 20 mM Tris–HCl (pH 7.5), 5 mM EDTA, 150 mM NaCl, 1% NP-40, 0.1% SDS, and 0.5% deoxycholate, with a protease inhibitor cocktail (Roche). Protein amounts in the cell lysates were determined using a Pierce bicinonic acid (BCA) protein assay following the manufacturer’s instructions (Thermo Fisher Scientific, Inc.). Equal amounts of proteins from each cell type were separated by electrophoresis on a 10% SDS-polyacrylamide gel, transferred to nitrocellulose membrane (GE healthcare), and processed as described previously [21]. GAPDH was used as the endogenous protein control and its specific mouse monoclonal antibody (sc-47724) was obtained from Santa Cruz Biotechnology Inc. Horseradish peroxidase (HRP) conjugates to mouse IgGκ light chain binding protein (Santa Cruz Biotechnology, Inc.), rabbit anti-goat IgG antibody (Thermo Fisher Scientific, Inc.), and swine anti-rabbit IgG antibody (Dako) were used as secondary antibodies. The immunoreactive proteins were visualized by SuperSignal West Pico PLUS enhanced chemiluminescence (ECL) substrate kit (Thermo Fisher Scientific, Inc.).

### NS1 capture ELISA

Each ELISA well was coated with 100 μl of 10 μg/ml of anti-NS1 antibody (clone 2E11). After blocking with 4% bovine serum albumin (BSA) in PBS, different concentrations of purified DENV-2 NS1 (0.16–25 ng/ml) and varied dilutions of samples (1:5, 1:25 and 1:125) were added to pre-coated wells and incubated for 1 h at 37°C. Biotinylated anti-NS1 antibody (clone 1B10) was added to the wells and incubated for 1 h at 37°C, followed by streptavidin-HRP (1:5,000 dilution, Sigma) for 1 h at 37°C. The plates were washed four times with PBST (PBS+0.1%Tween) between each step. After adding the tetramethylbenzidine (TMB) substrate (Thermo Fisher Scientific, Inc.), the colorimetric reaction products were measured at 450/620 nm in an ELISA plate reader (Biochrom Anthos 2010).

### Assessment of cell viability

The three cell lines were plated in 24-well plates (2 x 10^5^ cells/well) and cultured in their corresponding media overnight prior to infection with different MOIs (0.1, 1 and 5) of DENV-2. At 0, 6, 12, 24 and 48 h post-infection, the cells were detached from the wells by incubation in PBS containing 0.1% trypsin and 2.5 mM EDTA (3 min, 37°C), washed once with washing medium, and immediately assessed for viability as follows. Early and late apoptotic cells were determined using Annexin V-FITC/Propidium iodide (PI) staining kit (BD Biosciences) according to the manufacturer's instructions. The apoptotic cells were also analyzed based on caspase-3 and caspase-7 activation using CellEvent Caspase-3/7 Green detection reagent (Thermofisher Scientific Inc.), according to the manufacturer's instructions. The percentages of positively stained cells were determined by flow cytometry and the data were analyzed by Flowjo software.

### Analysis of cytokine gene and protein expression

The three cell lines and PHHs were plated in 24-well plates and cultured in their corresponding media overnight prior to mock infection or DENV-2 infection with different MOIs (0.1, 1 and 5) for 48 h, or with only MOI of 5 for 0, 6, 12, 24 and 48 h. The cells and culture supernatants were collected at the specified time points for evaluation of cytokine expression.

The levels of secreted cytokine molecules in the culture supernatants were measured using Bio-Plex Pro Human Cytokine 27-plex assay and a Luminex machine (Bio-Rad) according to the manufacturer’s instructions. The assay includes 27 different cytokines, namely interleukin (IL)-1β, IL-1rα, IL-2, IL-4, IL-5, IL-6, IL-7, IL-8, IL-9, IL-10, IL-12, IL-13, IL-15, IL17, interferon (IFN)-γ, IFN-γ-inducible protein (IP)-10, monocyte chemoattractant protein (MCP)-1, macrophage inflammatory protein (MIP)-1α, MIP-1β, Eotaxin, basic-fibroblast growth factor (Basic-FGF), granulocyte colony-stimulating factor (G-CSF), granulocyte macrophage colony-stimulating factor (GM-CSF), platelet-derived growth factor (PDGF), vascular endothelial growth factor (VEGF), RANTES, tumor necrosis factor (TNF)-α. Duplicates of the cultured supernatants (50 μl/replicate) of three hepatic cell lines and PHHs were used for the assay.

Gene expression profiles of different cytokines were determined using RT-PCR. Total RNA was extracted from cells (0.3–0.5 million cells/replicate) using Ambion TRIzol Reagent (Thermo Fisher Scientific Inc.) following the manufacturer’s instructions. Total extracted RNA was quantified using a NanoDrop 2000/2000c spectrophotometer (Thermo Fisher Scientific Inc.). Total RNA (500 ng/sample) was subjected to cDNA synthesis using AMV reverse transcriptase (Promega) and Oligo(dT)15Primer (Promega). Primer sequences specific for MCP-1, IL-8, RANTES, IFN-β, MIP-1α, MIP-1β and TNF-related apoptosis inducing ligand (TRAIL) were as previously described [2]. For IFN-α and 18S ribosomal RNA, the primer sequences are shown elsewhere [22]. Primer sequences for TNF-α (Forward 5′ GCC TCT TCT CCT TCC TGA TCG T 3′; Reverse 5′ GAG CTG CCC CTC AGC TTG 3′) were modified from the previous study [23]. Conventional PCR was conducted using Taq DNA polymerase (Promega) with 2 μl of cDNA and the amplification was performed for 35 cycles using the following conditions: denaturation at 95°C for 30 sec, annealing at 55°C for 1 min for all genes except MCP-1 (68°C), and extension at 72°C for 1 min. PCR products were separated on 2% (w/v) agarose gels and visualized subsequently by ethidium bromide staining.

### Inhibitory effects of drugs on DENV replication in different hepatocyte cell lines

HepG2, Huh-7 and imHC were plated in 24-well plates (2 x 10^5^ cells/well) and cultured overnight in their specific media. The cells were mock infected or infected with DENV-2 at MOI of 0.1 for 2 h, washed three times with plain media, and further cultured at 37°C for 48 h in their specific media in the presence or absence of ivermectin (5 μM solubilized in 0.5% DMSO) or ribavirin (10 μM solubilized in the culture medium). The 0.5% DMSO solution or medium was also used in place of drugs as control vehicles. After 48 h, the cells and supernatants were harvested to evaluate the inhibitory effects of drugs on DENV infectivity by determination of intracellular NS3 expression by flow cytometry, infectious virion production by FFU assay, and soluble NS1 secretion by ELISA as described above.

### Lipid droplet staining and analysis

For lipid droplet staining, hepatocytes (1 x 10^5^ cells/well) were cultured in 8-well chamber slides, and then infected with DENV-2 at a MOI of 0.1. Following 48 h of culture, the mock- or DENV-infected cells were fixed with 3.7% formaldehyde in PBS, washed once with PBS and incubated with BODIPY 493/503 (4, 4-difluoro 1, 3, 5, 7, 8 pentamethyl 4-bora 3a, 4a-diaza-s-indacene, Molecular Probes) at 2 μM for 15 min at 37°C. The cells were washed twice with PBS and nuclei were stained with Hoechst 33342 (1:1000 dilution, Thermo Fisher Scientific) at RT for 15 min before washing twice with PBS. Cells were viewed and images were obtained with a Carl Zeiss LSM800 with Airyscan confocal microscopy. The areas and number of lipid droplets were analyzed using Image J 1.50i software (http://imagej.nih.gov/ij).

### Lipid extraction and analysis by liquid chromatography combined with electrospray ionization/tandem mass spectrometry (LC-ESI-MS/MS)

HepG2, Huh-7 and imHC were cultured in 6-well plates (8 x 10^5^ cells/well) and mock-infected or infected with DENV-2 (MOI of 0.1). Following 48 h post-infection, the cells were detached by trypsinization, collected in microcentrifuge tubes and washed twice with PBS using low speed centrifugation to pellet the cells (300 x g for 5 min at 4°C). The cell pellets were resuspended in plain media and cell counting was performed using a hemocytometer to estimate the cell density. Aliquots containing equal numbers of each cell type were extracted using a modified Bligh and Dyer method [24].

Following a previously described system used for plasma lipid profiling with slight modifications [25], extracts corresponding to 2 million cells of each type (with or without DENV infection) were resuspended in 200 μl of a 1:1 mixture of solvent A (acetonitrile:water, 60:40, v/v, containing 10 mM ammonium formate) and solvent B (isopropanol:acetonitrile, 90:10, v/v, containing 10 mM ammonium formate). Ten μl was injected onto a reversed phase column (Waters Acquity BEH C_18_, 1.7 μm; 100 mm by 2.1 mm) equilibrated in 90% solvent A and 10% solvent B and eluted (100 μl/min) with an increasing concentration of solvent B (min/%B: 0/10, 5/50, 65/100, 70/5, 80/5). The effluent from the column was directed to an electrospray ion source connected to a triple quadrupole mass spectrometer (Waters Xevo TQ) scanning in the positive ion mode with previously optimized conditions [*m/z* from 200–1700 for phosphatidylcholines (PCs) and 500–1000 for triacylglycerols (TAGs), capillary voltage 3 kV, cone voltage 40 V, collision energy voltage 30 V, desolvation temperature 350°C, desolvation gas flow 650 L/h, nebulizer pressure 7 bar, and cone gas flow at 150 L/h]. Standards, 1,2-dipalmitoyl-*sn*-glycerol-3-phosphocholine [PC (16:0/16:0) or DPPC; Avanti Polar Lipids] and 1,2,3-triheptadecanoylglycerol [TAG (17:0/17:0/17:0); Sigma], were utilized for optimizing LC and MS parameters for profiling of PCs and TAGs, respectively. Precursor ion scans of *m/z* 184 were used for PCs, and neutral loss scans of 273, 271, 299 and 301 were used for TAGs containing C16:0, C16:1, C18:1, and C18:0 fatty acyl chains, respectively.

Data acquisition and peak area measurements were performed using instrument manufacturer-supplied software (MassLynx software version 4.1 SCN950; Waters). Relative abundances of TAGs were calculated from the areas of peaks in individual *m/z* ion traces from each neutral loss scan, normalized by the peak area of the peak in the *m/z* 734 ion trace corresponding to the [M+H]^+^ ion from DPPC in the precursor ion scan of *m/z* 184 of the same sample. The ratios to DPPC of selected TAG signals from the same neutral loss scan were combined and the sum of these ratios was used for comparison of relative TAG levels between mock- and DENV-infected conditions of each cell type (see **S1 Table** for details of selected TAGs used for analyses in this study).

### Statistical analysis

Two-way analysis of variance (ANOVA), followed by Bonferroni’s multiple comparison test were used to evaluate differences among cell lines infected with different MOIs of DENV-2 and/or at different time points. For the study of drug effects, multiple comparisons with Dunnett’s test were made between control and drug treatment groups for each cell type using R Statistical Software (version 3.6.3; R Foundation for Statistical Computing, Vienna, Austria). *P* values of less than 0.05 were considered to be significant for all tests performed. Unless stated otherwise, all statistical analyses were performed using GraphPad Prism 8 for OS X, Version 8.3.1 (332). The data analyzed were obtained from at least 3 independent experiments.

## Results

### Characterization of hepatocyte markers, DENV binding efficiency and DENV receptors in the hepatic cell models

Our previous study demonstrated the expression of liver-specific genes and proteins in imHC, indicative of a hepatic phenotype [9]. Herein, we further verified the protein expression and subcellular localization of selective hepatocyte markers in imHC compared to the two widely used hepatoma cell lines, HepG2 and Huh-7. Immunofluorescent staining revealed intense positive staining for hepatic markers, including human albumin (ALB), α-fetoprotein (AFP), low-density lipoprotein receptor (LDLR), multidrug resistance-associated protein 2 (MRP2), and hepatocyte nuclear factor 4α (HNF-4α) in the imHC and the other two cell lines, suggesting similar hepatic protein characteristics of these cell types (**S1 Fig**).

**Fig 1:**
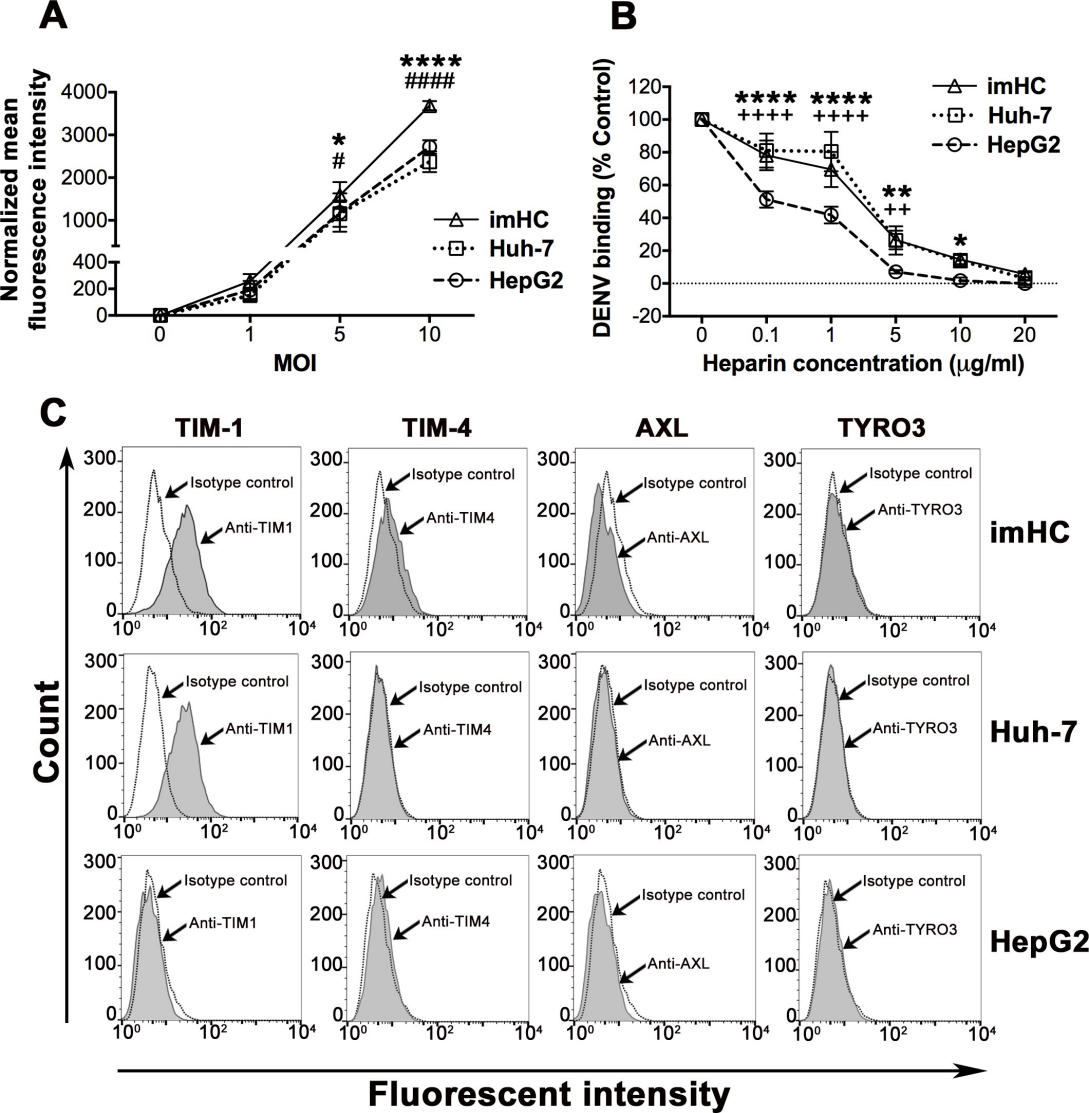
Characterization of DENV binding ability and potential DENV receptors of imHC in comparison with commonly used hepatoma cell lines (Huh-7 and HepG2). (**A**) The three hepatic cell types (imHC, Huh-7, HepG2) were incubated with different MOIs of DENV-2 for 1 h at 4°C, and the levels of DENV-2 bound to the cell surface were determined by immunofluorescent staining with anti-E antibody followed by flow cytometric analysis. In the absence of DENV, each cell type has unique background immunofluorescent staining. Therefore, the mean fluorescence intensity (MFI) values from the control (no DENV) condition of each cell type were subtracted from MFI values obtained from the corresponding cells in the presence of DENV. Data shown are mean ± S.D. of the normalized MFI values from three independent experiments. (**B**) The three hepatic cell types were incubated with DENV-2 at MOI of 5 for 1 h at 4°C in the presence of heparin at different concentrations, and the bound DENV levels were determined as described in **A**. The percentages of DENV binding to cells were calculated by comparing the subtracted MFI values of heparin-containing conditions to those from the control (no heparin) conditions of the same cell type. Data shown are mean ± S.D. of values from three independent experiments. An asterisk (*), “**#**” symbol and “**+**” symbol indicate significant differences between imHC vs HepG2, imHC vs Huh-7, and Huh-7 vs HepG2, respectively (* *P* < 0.05; ** P < 0.01; **** *P* < 0.0001). (**C**) Representative flow cytometry histograms showing signals on the surface of three cell types stained with specific antibodies for indicated DENV receptors (gray) compared with the isotype control.

Since binding of DENV to the host cell surface is the important initial step for infection, the viral binding efficiency of imHC was first examined and compared with the hepatoma cell lines. Various MOIs of DENV-2 were incubated with the three cell lines, and levels of DENV-2 binding to the cell surface were determined by positive staining of the DENV E protein. Flow cytometric results revealed that levels of DENV-2 attached to the surface of the three hepatic cell lines were similar at MOI of 1, but at MOIs of 5 and 10 imHC showed a significantly higher ability to bind to DENV-2 as compared to HepG2 and Huh-7 (**Fig 1A**).

Heparan sulfate proteoglycans (HSPGs) have previously been detected in hepatocytes of normal human liver tissues and hepatoma cell lines (HepG2) [26]. Roles of HSPGs and their heparan sulfate moieties in mediating initial binding of different viruses (i.e., hepatitis C and DENV) to hepatocytes have been shown [18,27–29]. To determine the role of heparan sulfate on DENV binding to imHC cells in comparison to that of the other cell lines, heparin, a structural analog of heparan sulfate [27], was used as a competitive inhibitor of DENV binding. DENV binding to the three cell types was inhibited by heparin in a concentration-dependent manner and viral binding to all cell types was abolished by heparin at a concentration of 20 μg/ml (**Fig 1B**). However, the inhibitory effects of heparin on DENV binding were significantly greater in HepG2 as compared to imHC and Huh-7 at the same heparin concentrations (0.1 to 10 μg/ml) (**Fig 1B**). These results suggest that expression levels of HSPGs on HepG2 might be different from imHC and Huh-7. Supporting this observation, our Western blotting result revealed lower levels of syndecan-1 –the primary HSPG of hepatocytes with important roles in viral binding and hepatic clearance [29,30], in HepG2 as compared to those of imHC and Huh-7 as well as PHHs (used as a positive control for syndecan-1 expression) (**S2A Fig**). Of note, syndecan-1 appeared as multiple bands in the range of 80–200 kDa due to the heterogeneity in size and number of heparan sulfate chains, as previously observed in other studies [31,32].

To determine whether DENV binding to hepatocytes relies on DENV receptors other than HSPGs, we further determined the expression of previously reported DENV receptors on mammalian cells, including those in the TIM and TAM (Tyro3-Axl-Mer) families [33], on the surface of these hepatic cell lines. Interestingly, while TIM-1 was expressed on the surfaces of imHC, Huh-7 and a positive control HEK-TIM1 cells, the protein was not detectable on the plasma membrane of HepG2 and PHHs (**Figs 1C, S2F and S3A**). Immunoblotting analysis showed that PHHs and HepG2 indeed expressed TIM-1 protein, but its form was different from that expressed in imHC and Huh-7. Under reducing conditions, TIM-1 appeared at 50 and ~100 kDa in imHC, Huh-7 and HEK-TIM1 cells, but was found as a high molecular weight band (~200 kDa) in PHHs and HepG2 under non-reducing conditions (**S2B Fig**). This difference is likely related to the lack of TIM-1 expression on the surface of PHHs and HepG2 (**Figs 1C and S3A)**. Although TIM-4, Axl and Tyro3 have been reported as DENV receptors on the surface of different cell types [33,34], they were not detectable on the surface of hepatocytes used in this study (**Fig 1C**) and their absence in hepatic cell lines and PHHs was confirmed by immunoblotting (**S2 Fig**).

To further assess the contribution of TIM-1 as an entry receptor for DENV in human hepatocytes, TIM-1-specific siRNA (siTIM1) knockdown experiments were performed in imHC. A reduction of ~50% in surface TIM-1 expression levels (**S3B Fig**) did not affect DENV infection and production in siTIM1-treated cells (**S3C and S3D Fig**), indicating that TIM-1 did not function as a primary entry receptor for DENV in imHC.

### Efficiency of DENV replication and production in hepatocyte models

**Fig 2:**
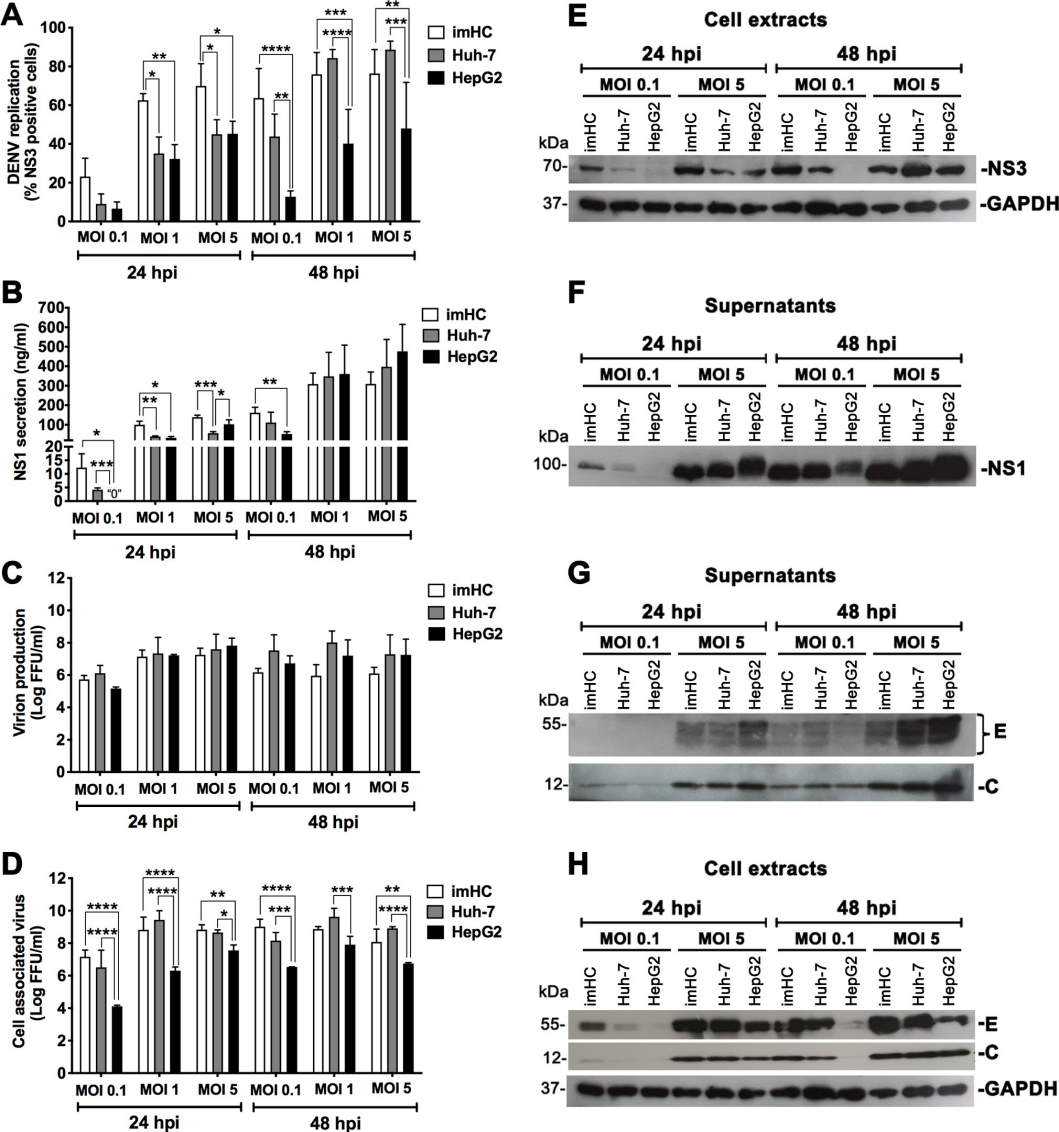
Efficiency of imHC in DENV replication and production in comparison with hepatoma cell lines. The three hepatic cell lines were cultured in 24-well plates and infected with different MOIs of DENV-2 (0.1, 1 and 5). The cells and the culture supernatants were collected at 24 and 48 hpi. Intracellular expression levels of NS3, a DENV antigen representing DENV replication, were determined by immunofluorescence and flow cytometry (**A**). NS1 secretion levels were determined by ELISA (**B**). The infectious virion production levels in supernatants (**C**) and cell-associated viruses in cell extracts (**D**) were assessed by FFU assay. The data are presented as mean ± S.D. of values from three independent experiments. Asterisks indicate significant differences among cell types at the specified time point (* *P* < 0.05; ** P < 0.01; *** P < 0.001; **** *P* < 0.0001). Levels of intracellular NS3 (**E**), secreted NS1 (**F**), extracellular virion E and C proteins (**G**) and cell-associated DENV E/C antigens (**H**) were evaluated by Western blotting analysis. Cell lysates containing equal amounts of proteins (15 μg/sample) or equal volumes of supernatants (10 μl/sample) were used for Western blotting. GAPDH was used as endogenous protein control for cell lysates.

To further verify whether imHC could serve as a suitable host cell model for DENV infection, we infected imHC with DENV-2 at various MOIs and determined its efficiency in supporting DENV replication and production at 24 and 48 h post infection (hpi), in comparison with HepG2 and Huh-7. DENV replication was evaluated based on expression of intracellular NS3, a key DENV nonstructural protein with dual functions in viral replication (i.e., protease and RNA helicase [35,36]). At 24 hpi, the percentage of cells positive for NS3 in imHC populations were significantly higher than those in Huh-7 and HepG2 at MOIs of 1 and 5. At 48 hpi, both imHC and Huh-7 showed significantly higher percentages of NS3-positive cells than HepG2 cells (**Fig 2A**). These differences in NS3 expression levels among cell types were confirmed by Western blotting analysis (**Fig 2E**).

We also assessed the levels of DENV non-structural protein 1 (NS1), a glycoprotein that is absent from viral particles, but is secreted from infected cells and plays roles in disease pathogenesis [37]. Analogous to the NS3 immunostaining results, NS1 ELISA results showed that imHC secreted NS1 at significantly higher levels than the other two cell lines at 24 hpi (i.e., MOIs of 0.1 and 1 for HepG2, and MOIs of 1 and 5 for Huh-7); this significant difference between NS1 levels of imHC and HepG2 was also observed with MOI of 0.1 at 48 hpi (**Fig 2B**) and further confirmed by Western blotting analysis of NS1 in the culture supernatants (**Fig 2F**). Nevertheless, levels of infectious virion production in the culture supernatants were not significantly different among the three cell types in all tested conditions, as shown by FFU assay (**Fig 2C**) and Western blotting of DENV structural proteins (**Fig 2G**). Interestingly, unlike virion production levels, the amount of intracellular infectious virions (**Fig 2D and 2H**) correlated with both NS3 expression and NS1 secretion levels in all three hepatic cell types. Together, these findings indicate that the efficiency of imHC in supporting DENV replication and NS1 secretion was greater than the commonly used hepatoma cell lines, particularly HepG2.

**Fig 3:**
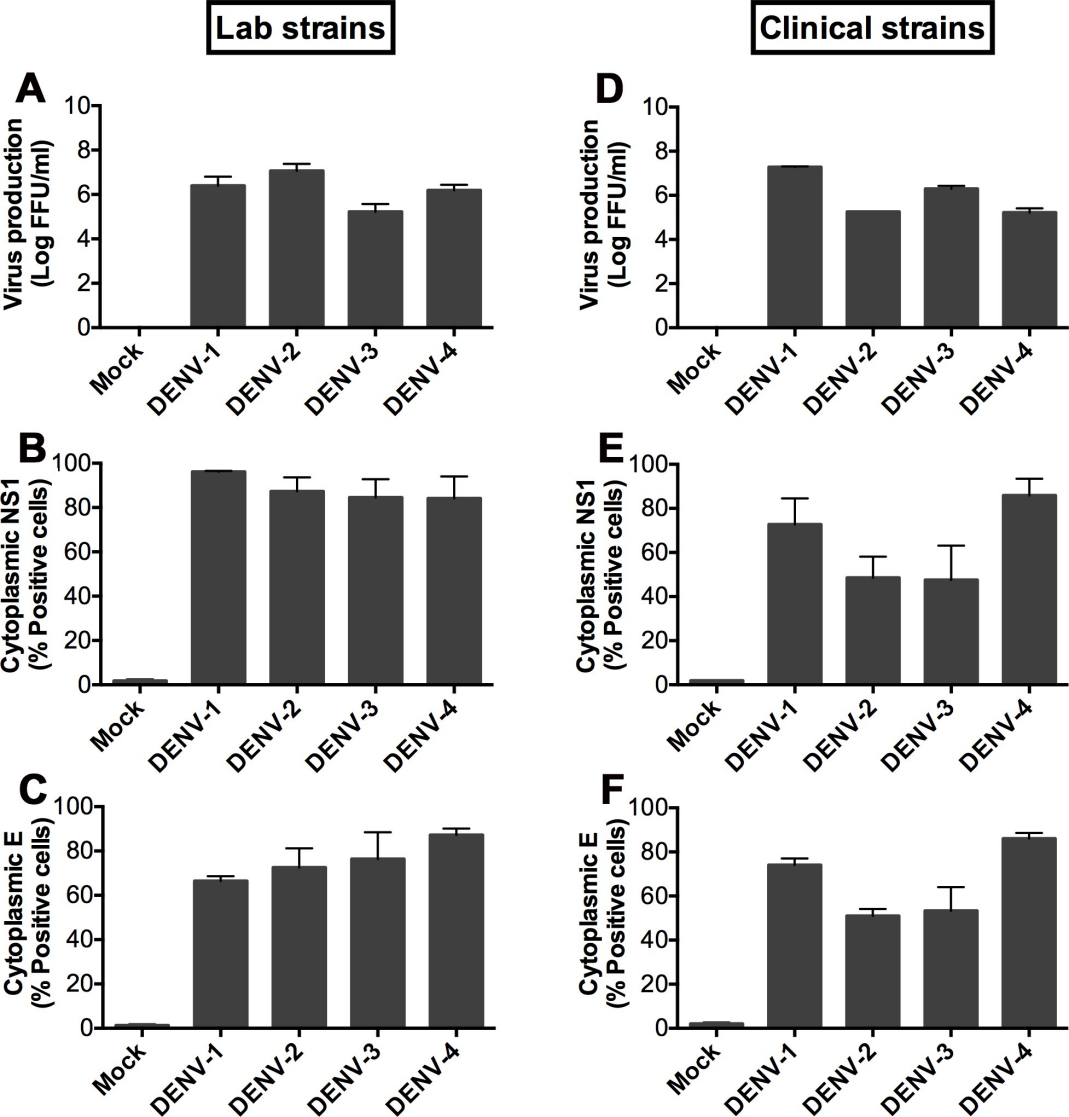
Productive infection of all four DENV serotypes, both laboratory and clinically isolated strains, in imHC. imHC were infected with four different DENV serotypes (DENV-1, DENV-2, DENV-3 and DENV-4) of either laboratory (**A**, **B** and **C**) or recently isolated clinical strains (**D**, **E** and **F**) at MOI of 0.1 for 48 h. The cell culture supernatants were subjected to the FFU assay to determine the levels of viral production (**A** and **D**). The percentages of DENV-infected cells were analyzed by immunofluorescent staining of cytoplasmic NS1 (**B** and **E**) and envelope (**C** and **F**) proteins, followed by flow cytometry. Data are presented as mean ± S.D. of values from three independent experiments.

Besides DENV-2 (16681 lab strain) used mainly in this study, we determined whether imHC could support infection by other DENV serotypes and strains. Importantly, imHC were susceptible to both lab and clinical strains of all four DENV serotypes and could produce comparable levels of DENV antigens and virions upon infection by different DENV strains and serotypes (**Fig 3**).

### Cell viability of hepatocyte cell lines during the time course of DENV infection

**Fig 4:**
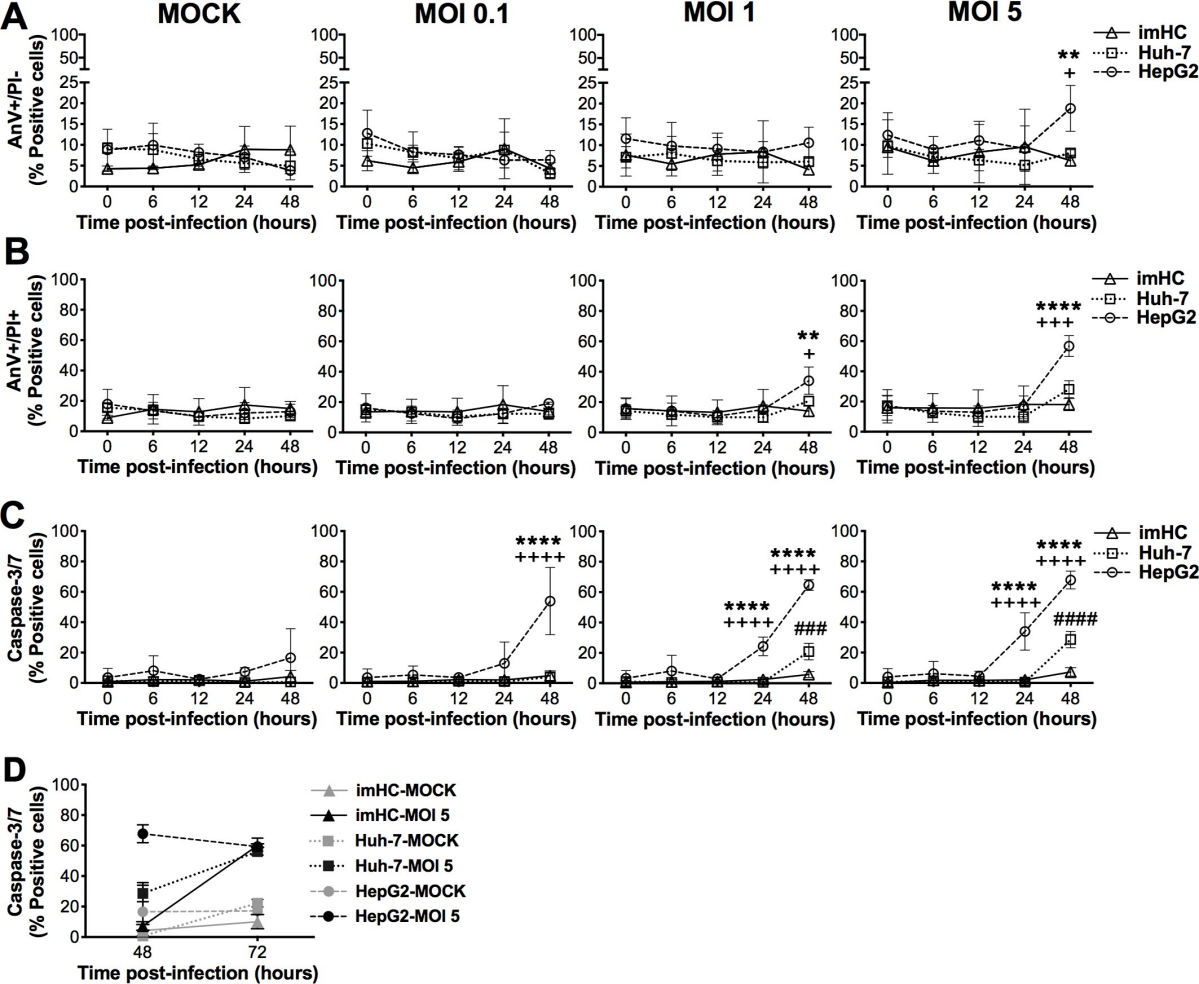
Viability of imHC and the hepatoma cell lines over the time course of DENV-2 infection. The cells were cultured in 24-well plates and mock-infected or infected with different MOIs of DENV-2 (0.1, 1 and 5), and then assessed for their viability at the indicated time points post-infection. (**A**) Graphs showing percentages of cells that had positive staining for annexin V but negative staining for propidium iodide (AnV+/PI-) (early apoptotic cells). (**B**) Graphs showing percentages of cells that were positively stained for both AnV and PI (AnV+/PI+) (late apoptotic cells). (**C-D**) Graphs showing percentages of apoptotic cells with positive staining for caspase-3/7 activation. All data are presented as mean ± S.D. of values from three independent experiments. An asterisk (*), “**#**” symbol and “**+**” symbol indicate significant differences between imHC vs HepG2, imHC vs Huh-7, and Huh-7 vs HepG2, respectively (* *P* < 0.05; ** *P* < 0.01; **** *P* < 0.0001).

DENV infection has been shown to associate with apoptosis of hepatocytes both *in vitro* and *in vivo* [3,38,39]. We first determined levels of apoptosis after DENV exposure to all three hepatic cell types using Annexin V (AnV)/propidium iodide (PI) staining. At a MOI of 0.1, no differences in early (AnV^+^/PI^-^, **Fig 4A**) or late (AnV^+^/PI^+^, **Fig 4B**) apoptotic cell numbers were observed between mock and DENV-infected cultures of all three cell lines. However, at higher MOIs (1 and 5), a significant rise in early (**Fig 4A**) and late (**Fig 4B**) apoptotic cells was found in DENV-infected HepG2 at 48 hpi. By employing a more sensitive assay to detect intracellular caspase-3/-7 activity, the two important cysteine proteases activated during the early stage of apoptosis [40,41], the results confirmed elevated apoptosis in HepG2 cultures exposed to DENV (even at a low MOI of 0.1) which became detectable at 24 hpi and were more pronounced at 48 hpi (**Fig 4C**). Notably, DENV also induced apoptosis in Huh-7 cultures, especially under conditions of high viral burden (MOIs of 1 and 5, **Fig 4C**). Surprisingly, apoptosis was not detectable in the cultures of imHC up to 48 h after DENV exposure (**Fig 4A–4C**). However, a rise to ~50% apoptotic cells in both imHC and Huh-7 was noted at 72 h post infection with DENV-2 at MOI of 5 (**Fig 4D**). Overall, these data suggest differential apoptotic responses upon DENV infection among the three hepatic cell types; HepG2 are most susceptible whereas imHC are the most resistant to DENV-induced apoptosis.

### Cytokine protein and gene expression of hepatocytes in response to DENV infection

**Fig 5:**
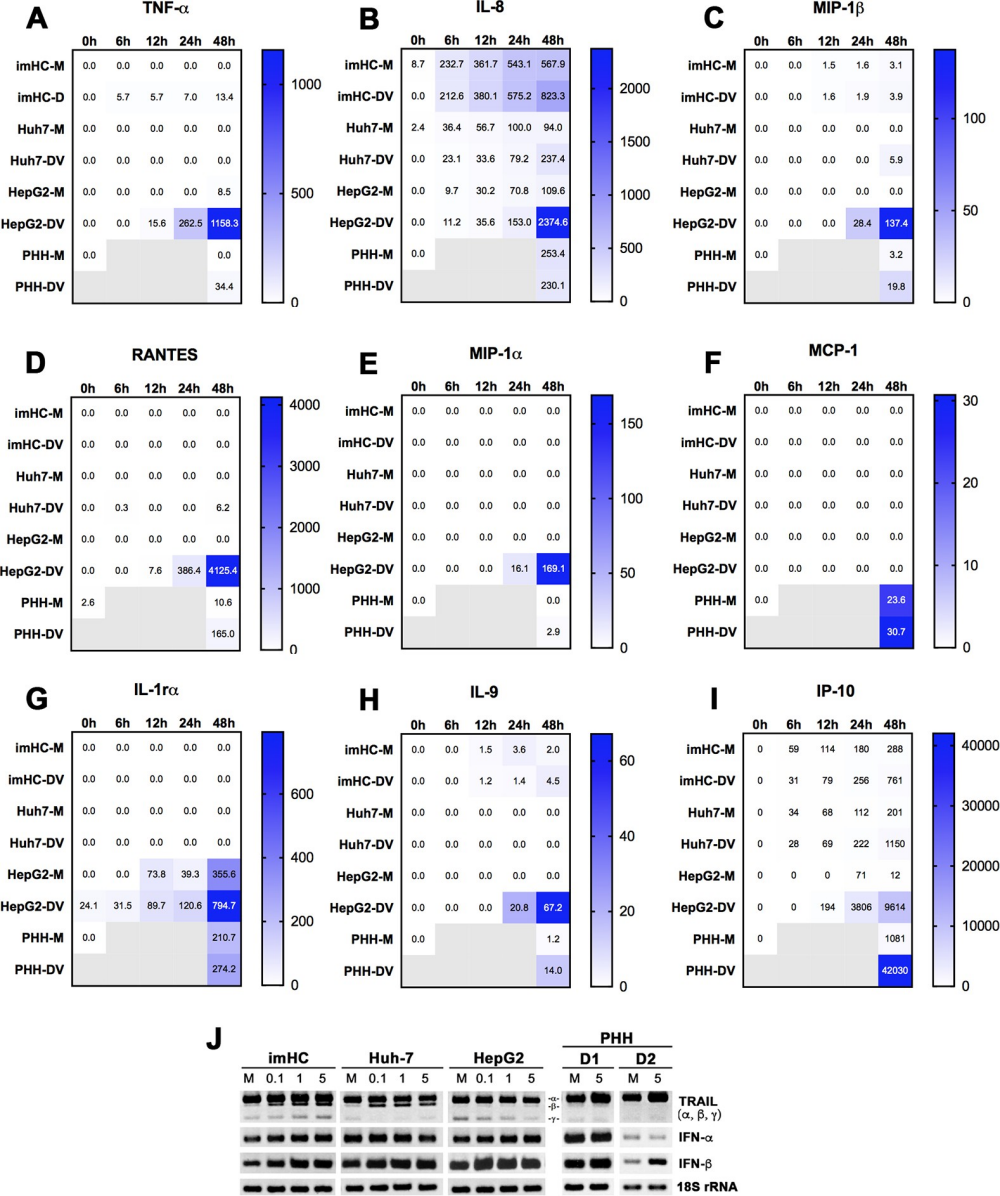
Cytokine expression of hepatocytes upon DENV-2 infection. The three hepatic cell lines and PHHs from 2 different donors (D1 and D2) were mock-infected (M) or infected with DENV-2 (DV) at MOI of 5. At the specified time points, cells and culture supernatants were harvested for assessment of cytokine expression. (**A-I**) A heatmap of differential cytokine protein production by hepatic cell lines and PHHs was quantified by Bio-Plex Pro Human Cytokine 27-plex assay. The numbers shown in the heatmap are average amounts (pg/ml) of secreted cytokines from two experiments of hepatic cell lines or two donors for PHHs. An equal number of each cell type was used for RNA extraction and an equal amount of RNA from each source was further subjected to RT-PCR and gel electrophoresis. The three hepatic cell lines and PHHs from 2 different donors (D1 and D2) were mock-infected (M) or infected with different MOIs (0.1, 1 and 5) of DENV-2. The cells were harvested for RT-PCR analysis of TRAIL, IFN-α, and IFN-β and a representative gel, showing RT-PCR products corresponding to these cytokines, is shown in **J**.

A cytokine storm is a pathogenic factor that contributes to severe dengue infections [41]. Since hepatocytes are primary target cells for DENV replication *in vivo* [15,42] and are known for their ability to produce cytokines in response to flavivirus infections [43,44], the kinetics of cytokine gene and protein expression after DENV infection were evaluated and compared among the hepatic cell lines and PHHs (**Fig 5**). Productive infection of PHHs from two donors was confirmed prior to the assessment of cytokine expression patterns (**S4 Fig**).

Among 27 cytokine proteins included in this study, 9 cytokines (TNF-α, IL-8, MIP-1α, MIP-1b, MCP-1, RANTES, IL-1rα, IL-9, IP-10) were found at measurable levels in culture supernatants of PHHs following mock infection and/or DENV-2 infection at MOI of 5 for 48 h (**Fig 5A–5F**). Expression of 6 cytokines in PHHs were further confirmed at the gene level (**S5 Fig**), whereas RT-PCR of the other 3 cytokines (IL-1rα, IL-9, IP-10) was not performed in this study (**Fig 5G–5I**).

The protein secretion and/or gene expression levels of these 9 cytokines in responses to kinetics of DENV infection varied among the three cell lines and PHHs. Upon DENV infection, TNF-α was detectable at protein and gene levels in imHC, HepG2 and PHHs, but only observed at the gene level in Huh-7 (**Figs 5A and S5**). Interestingly, unlike TNF-α, IL-8 was constitutively expressed at both gene and protein levels in all cell lines and was induced following DENV infection, especially at later time points (24 and 48 h) (**Figs 5B and S5**). However, DENV-induced IL-8 expression was not clearly observed in PHHs (**Fig 5B**). The increase in MIP-1β upon DENV infection, particularly at 48 hpi was detected at both protein and gene levels in all three cell lines and PHHs (**Figs 5C and S5**). RANTES secreted protein was found in Huh-7, HepG2 and PHHs, although the gene expression and up-regulation upon DENV infection at 48 hpi were detectable in all cell types (**Figs 5D and S5**). Similar to RANTES, DENV-induced up-regulation of MIP-1α (at 48 hpi) was detected in all cell types at the gene level (**S5 Fig**). However, secreted MIP-1α protein was only measurable in HepG2 and PHHs (**Fig 5E**). Unlike PHHs, MCP-1 secretion was not detectable in any cell line (**Fig 5F**); however, its induction following 48h-DENV infection was observed at the gene level in imHC (**S5 Fig**). Similar to MIP-1α, secreted IL-1rα was only detected in HepG2 and PHHs and its levels were increased upon DENV infection (**Fig 5G**). Similar to TNF-α, IL-9 was detectable in all cell types, except Huh-7 and its level was up regulated by DENV infection in HepG2, imHC and PHHs (**Fig 5H**). Significantly, IP-10 was measurable at high levels (e.g., ~30–40000 pg/ml) and showed increased levels by DENV induction in all cell lines and PHHs (**Fig 5I**). Notably, unusually high levels of all secreted cytokines in HepG2 were measurable especially at later time points (24 and 48 h) after DENV infection were likely caused by a significantly higher degree of apoptotic cell death in HepG2 (**Fig 4**).

In addition to the cytokine molecules listed above, we determined gene expression levels of TRAIL, IFN-α, and IFN-β, previously shown in hepatocytes in responses to DENV infection [45]. Various isoforms of TRAIL from alternative RNA splicing [46], including full-length TRAIL-α containing 5 exons (top band), truncated TRAIL-β lacking exon 3 (middle band) and truncated TRAIL-γ lacking exons 2 and 3 (bottom band), were differentially expressed in different lines of hepatocytes (**Fig 5J**). The apoptosis inducer TRAIL-α, a major TRAIL variant [46], was similarly detected in all cell types before and after DENV infection. TRAIL-β, a negative regulator of TRAIL-α showed a trend to increase after DENV infection in all cell types (**Fig 5J**). Remarkably, the concentrations of TRAIL-γ, another negative apoptosis regulator, were decreased in a dose-dependent manner by DENV infection in HepG2 but, on the other hand, increased in imHC (**Fig 5J**). This may partly explain the increased resistance of imHC to DENV-induced apoptosis (**Fig 4**). Following DENV infection, the IFN-α gene showed no obvious changes, whereas IFN-β had increased levels in all cell lines and PHHs from 2 different donors.

Overall, our results revealed that the hepatic cell lines and PHHs had similarities and dissimilarities in cytokine production. Upon DENV infection, relatively high amounts of IL-8, MIP-1β, IP-10 were secreted from all three cell lines and PHHs. The other cytokines secreted by PHHs were either absent from one type (TNF-α, IL-9 and RANTES), two types (MIP-1α and IL-1rα) or all types (MCP-1) of hepatic cell lines.

### Alterations in lipid droplets and triacylglycerol levels of hepatic cell lines upon DENV infection

**Fig 6:**
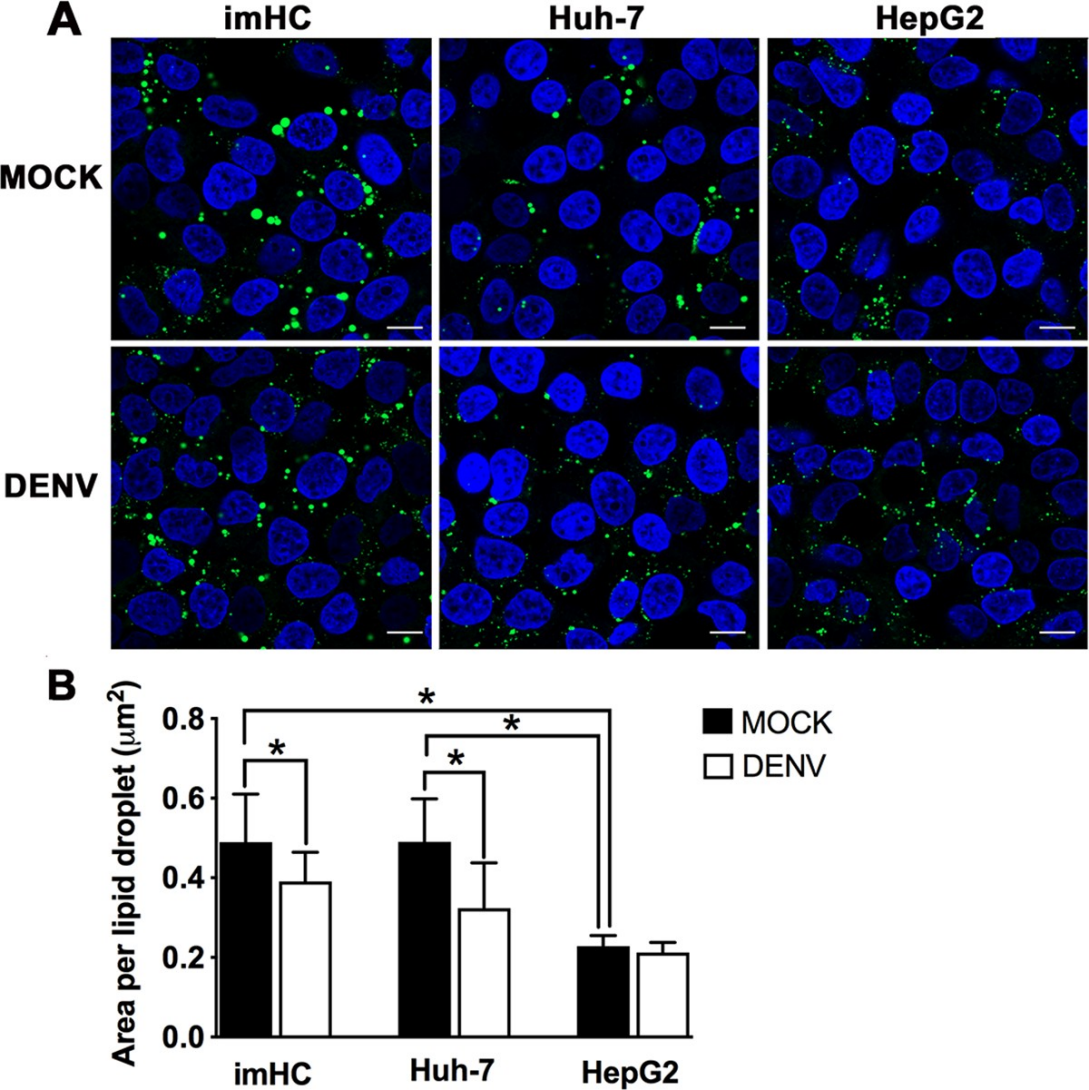
Changes in lipid droplet areas in imHC and hepatoma cell lines upon DENV infection. (**A**) Representative images from the confocal microscopic analysis of lipid droplets stained with BODIPY 493/503 in the three cell lines cultured for 48 h under mock-infected (top panel) or DENV-infected conditions (MOI of 0.1) (bottom panel). Nuclei were stained with Hoechst 33342. Scale bar = 10 μm. (**B**) Area per lipid droplet of each cell type pre- and post-DENV infection was further analyzed using Image J software. The data shown are the mean ± S.D. values of the areas of lipid droplets analyzed from a total of 300–400 cells for each cell type. Asterisks indicate statistically significant differences (*P* < 0.05) of area per lipid droplet among the three cell types pre-infection as well as between the mock and DENV-infected conditions of each cell type.

Lipid droplets are endoplasmic reticulum-derived lipid-rich organelles with important roles in lipid homeostasis. Accumulating lines of evidence indicate that they are modified in flavivirus-infected cells via lipophagy in order to facilitate viral production [47,48]. Hepatocytes are metabolically active cells that play an important role in controlling lipid droplet function and metabolism [49], and they have been linked to liver pathologies upon flavivirus infection [15,50]. To understand the impact of DENV infection on lipid metabolism of hepatocytes, we compared changes of lipid droplet abundance and composition in three hepatic cell lines following 48 h of DENV infection at an MOI of 0.1. Confocal microscopic analysis revealed different sizes of lipid droplets among the three cell types before and after DENV infection (**Fig 6A**). Further quantitative analysis using imageJ software revealed that imHC and Huh-7 had significantly larger areas of lipid droplets than HepG2 under mock conditions (**Fig 6B**). Moreover, following DENV infection, imHC and Huh-7 showed significant decreases in lipid droplet area, while HepG2 revealed no such changes (**Fig 6B**), suggesting distinct rates of lipid droplet metabolism in these cell lines. It is likely that molecular factors involved in lipid droplet enlargement and homeostasis are more active in imHC and Huh-7 than in HepG2.

**Fig 7:**
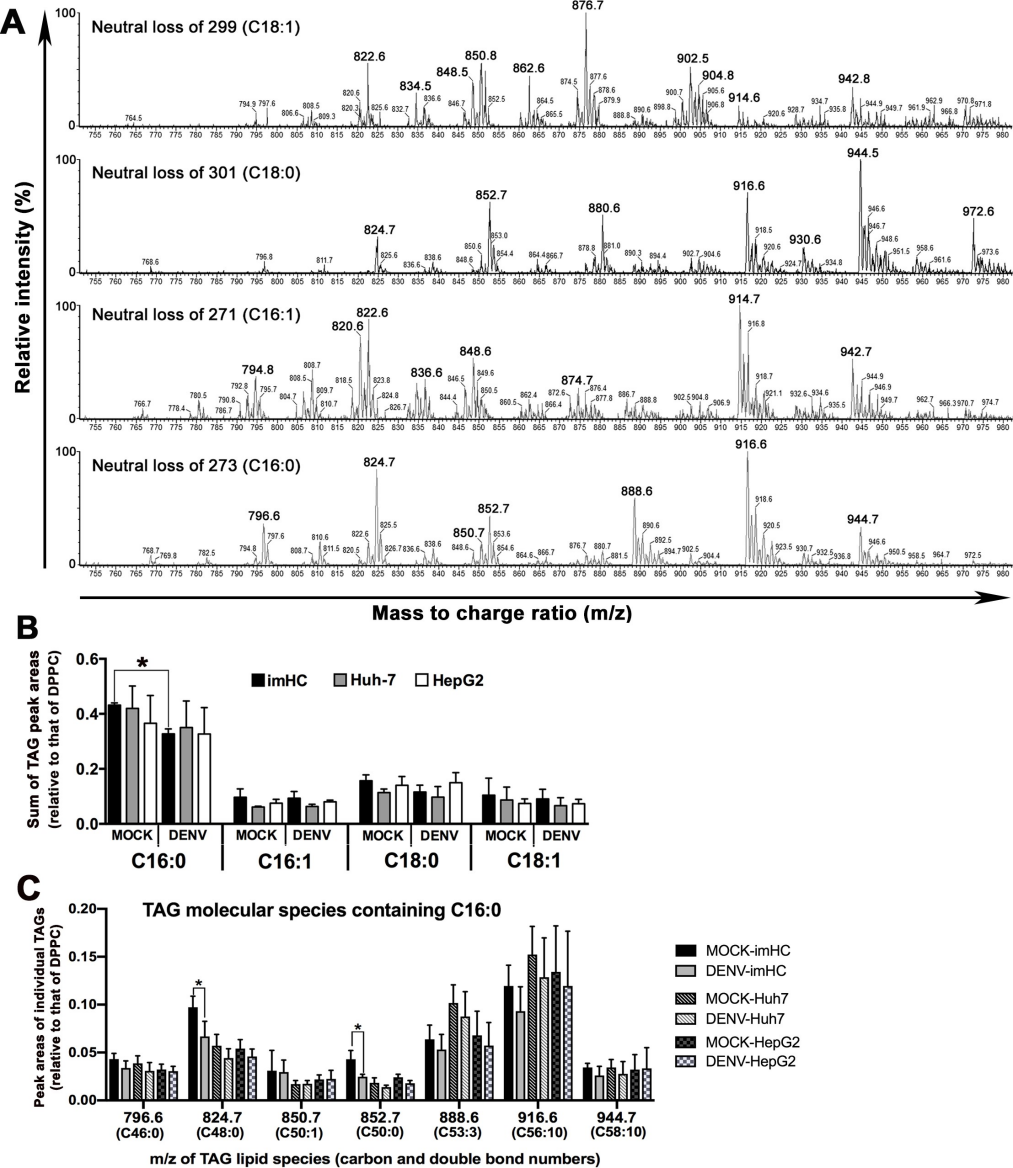
LC-ESI-MS/MS analyses for quantification of triacylglycerols (TAGs), major components of lipid droplets, in three hepatic cell lines before and after DENV infection. (**A**) Multiple neutral loss scans of ammoniated TAGs in hepatocyte lipid extracts. Representative MS/MS spectra from neutral loss scanning of 273, 271, 299, and 301, corresponding to the four common fatty acyl chains with C16:0, C16:1, C18:1, and C18:0, respectively, of the imHC extract. The major *m/z* signals from each scan (indicated by the larger *m/z* labeling) were selected for TAG species assignment, and quantification of the relative abundances of TAGs with different fatty acyl compositions (see **S1 Table** for details). (**B-C**) Changes of TAG relative abundances in the three hepatic cell lines upon DENV infection. Relative abundances of TAGs were quantified based on the peak areas of individual *m/z* signals from each neutral loss scan and normalized by the peak area of *m/z* 734 (DPPC–a major PC) in the same sample. The ratios to DPPC of selected TAG signals from the same neutral loss scan were combined and the sum of these ratios was used for comparison of TAGs containing different types of fatty acids (C16:0, C16:1, C18:0 and C18:1) between mock- and DENV-infected conditions of each cell type (**B**). The relative abundances of individual molecular species of TAGs with C16:0 in the three hepatic cell lines upon DENV infection are also shown (**C**). Data shown in B and C panels are the mean ± S.D. of values obtained from three sets of biological samples. Asterisks indicate statistically significant changes (*P* < 0.05) of TAG levels in imHC after DENV infection.

To gain insights into biochemical changes in lipid metabolism of the three hepatocyte cell lines upon DENV infection, we further determined lipid levels in whole hepatic cell extracts using mass spectrometry with the focus on triacylglycerols (TAGs), major components of lipid droplets [51]. Neutral loss scans were performed to detect TAGs containing the four most common fatty acyl chains, including palmitic (C16:0), palmitoleic (C16:1), oleic (C18:1), and stearic (C18:0) [52]. **Fig 7A** shows a representative mass spectral profile from the neutral loss scanning of imHC extracts in the *m/z* range of 750–980, a mass range that covers all major TAGs [53,54]. The TAG profiles from the four neutral loss scans were similar among the three hepatic cell lines and PHHs but different from those of non-hepatic cell types (U87-MG and PBMCs), especially for TAG species with C16:0 and C18:0 (**S6** and **S7 Figs**), suggesting that there is a characteristic TAG profile specific to hepatocytes. The *m/z* values in the spectra were used to assign probable identities of ammonium TAG adducts based on the LIPID MAPS database (www.lipidmaps.org). The details of TAG species detected in this study are shown in **S1 Table**. Major molecular species of TAGs found in each neutral loss scan (labeled in large font in **Fig 7A**) were selected for further comparison of their relative abundances in the three cell lines upon DENV infection. In this study, we compared abundances of TAGs based on their peak areas relative to that of dipalmitoylphosphatidylcholine [DPPC or PC (C16:0/16:0); *m/z* 734], a major phospholipid that exists in the three cell lines and shows no change upon DENV infection (**S8 Fig**). The overall relative abundances of TAGs, containing either C16:0, C16:1, C18:0 or C18:1, were similar among the three hepatic cell types prior to DENV infection (**Fig 7B**). However, following DENV infection (MOI of 0.1, 48 h), only imHC revealed a statistically significant change (a decrease of 25%) in the total abundances of C16:0 TAG species compared to the mock-infected cells (**Fig 7B**). When individual molecular species of TAGs with C16:0 were analyzed, *m/z* 824.7 TAG (TAG-C48:0 or C16:0/16:0/16:0) and *m/z* 852.7 TAG (TAG-C50:0 or C16:0/16:0/18:0) in imHC were the species that showed significant reduction upon DENV infection (**Fig 7C**); Structures of these TAGs are shown in **S9 Fig**. All of these results indicated that the TAG catabolism of imHC was more responsive to DENV infection than that of the hepatoma cell lines.

### Efficiencies of different hepatic cell lines in response to drugs with anti-DENV activity

Another advantage of using hepatocyte cell lines is their suitability for studying the effects and mechanisms of action of drugs. In this study, we chose two drugs (ribavirin and ivermectin) with known effects on virus replication. Ribavirin is a guanosine analog with broad-spectrum antiviral activity and has been a standard treatment regimen for hepatitis C virus (HCV). One of its known mechanisms is to inhibit *de novo* biosynthesis of guanine nucleotides, resulting in inhibition of viral RNA synthesis [55,56]. Ribavirin effects on DENV replication have also been shown [57]. Ivermectin is an FDA-approved anthelmintic drug that has recently been shown to have affinity for viral proteins and/or inhibitory effects on replication of DENV and other types of viruses (e.g., HIV, VEEV, CHIKV, ZIKV) [58–63]. Moreover, the results of our recently closed randomized placebo-controlled trial of ivermectin for treatment of adult dengue patients confirmed its *in vitro* virological efficacy in accelerating circulating NS1 clearance (ClinicalTrials.gov: NCT02045069; Suputtamongkol *et al*., forthcoming). The detailed pharmacokinetic and pharmacodynamic study of ivermectin in children infected with DENV to determine the appropriate dosage regimens is ongoing (ClinicalTrials.gov: NCT03432442).

**Fig 8:**
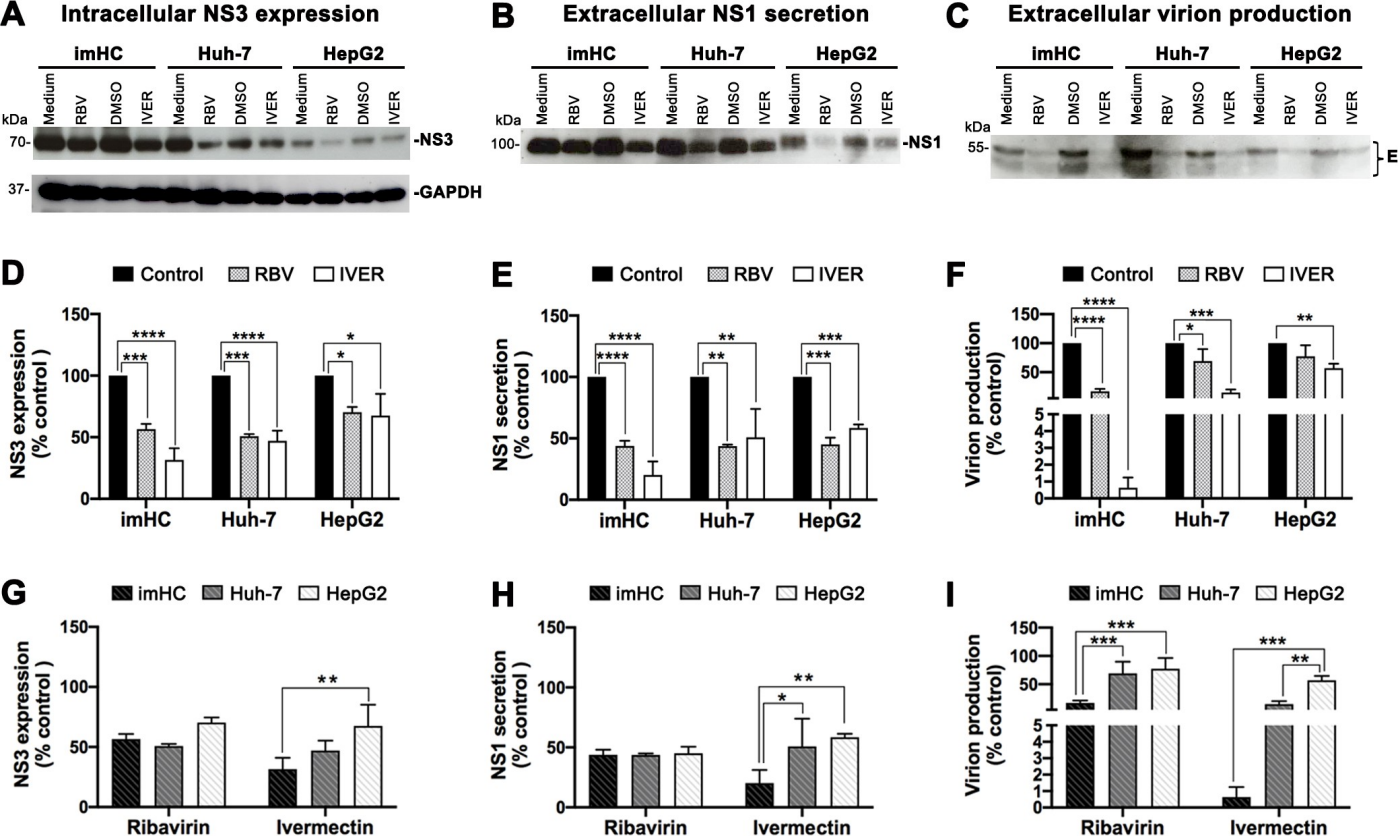
Differential inhibitory effects of anti-viral drugs on DENV replication in three hepatic cell lines. The three cell lines (HepG2, Huh-7, imHC) were infected with DENV-2 at MOI of 0.1 and cultured in media with or without drugs [5 μM of ivermectin (IVER) or 10 μM of medium-soluble ribavirin (RBV)]. Plain medium or medium containing 0.5% DMSO (used for ivermectin solubilization) was also used as control vehicles for ribavirin and ivermectin treatment, respectively. At 48 h post infection, the cells and culture supernatants were collected for assessment of drug effects on viral production and DENV antigen expression as follows: (**A-C**) Levels of indicated DENV antigens were determined by Western blotting analysis and GAPDH was used as an internal control protein, (**D** and **G**) Percentages of cells with cytoplasmic NS3 expression determined by flow cytometry, (**E** and **H**) NS1 secretion levels in culture supernatants quantified by ELISA, (**F** and **I**) Virion levels in culture supernatants determined by FFU assay. The data from drug treatment conditions of different cell types were calculated as percentages of their corresponding control vehicles. All data are presented as mean ± S.D. of values from three independent experiments. Asterisks indicate significance differences between conditions or cell types (* *P* < 0.05; ** *P* < 0.01; *** *P* < 0.001; **** *P* < 0.0001).

We first tested various drug concentrations to determine the 50% cytotoxicity concentration (CC_50_) and half maximum effective concentration (EC_50_) values for all three hepatic cell lines (**S2 Table**). The non-cytotoxic concentrations of 5 and 10 μM were chosen for ivermectin and ribavirin, respectively, for cell treatment in this study. We have also confirmed that drug treatment at the selected concentrations did not cause cell death in the three hepatic cell lines upon DENV infection (**S3 Table**). Following DENV infection (MOI of 0.1) and drug treatment (48 h), percentages of DENV-infected cells based on NS3 expression, levels of NS1 secretion and virion production were evaluated and compared among the three cell types (**Fig 8**). Western blotting analysis revealed that ribavirin and ivermectin treatment resulted in reduced levels of NS3 expression (**Fig 8A**), NS1 secretion (**Fig 8B**) and extracellular virion E (**Fig 8C**) in the three cell lines. The significant reduction in NS3-positive cells and secreted NS1 levels upon drug treatment were confirmed by flow cytometry (**Fig 8D**) and ELISA (**Fig 8E**), respectively. However, the significant effects on infectious virion production by both drugs were only observed in imHC and Huh-7, whereas statistically significant decrease in virion production of HepG2 was observed only with ivermectin treatment (**Fig 8F**). These results are in line with the kinetics results (**Fig 2**), indicating a better correlation between levels of intracellular NS3 and secreted NS1 than that of the virion production.

We further compared degrees of inhibitory effects of both drugs among the three cell types. For NS3 expression levels, similar effects of rivabirin were observed in all cell types, but imHC showed significantly greater degrees of reduction than HepG2 after ivermectin treatment (**Fig 8G**). The significant differences in reduction of NS1 secretion were also observed between imHC and the hepatoma cell lines upon ivermectin treatment (**Fig 8H**). Notably, imHC showed decreases in virion production levels in response to both drugs at significantly higher levels than in the hepatoma cell lines (**Fig 8I**). Significant inhibitory effects of ivermectin on DENV production of PHHs were also confirmed in this study (**S10 Fig**). All of these findings suggest that imHC serves as a superior model to hepatoma cell lines for further drug screening and detailed studies of mechanisms of anti-viral drug action.

## Discussion

Liver diseases, particularly hepatotropic infectious diseases, are major global health issues. The development of effective therapeutic strategies depends on the availability of disease models. Due to the cost and technical challenges associated with animal and primary cell models, the early stage of drug screening still relies on *in vitro* hepatic cell models. Although the hepatocarcinoma cell lines have been widely used for preclinical drug testing, their aberrant cell growth and metabolism raise concerns regarding their suitability in representing normal pathophysiologic features of human hepatocytes. Our present study indicates that the stem cell-derived imHC offer a promising alternative *in vitro* model, specifically for DENV, to hepatocarcinoma cell lines with regard to mechanistic insights of viral pathogenesis and anti-viral drug effects in human liver. Besides DENV, imHC have recently been shown to also serve as a suitable model for studying hepatitis B virus and malarial infection [64,65].

Several factors (e.g., surface expression of specific entry receptors, the viral-specific antagonism of host cytoplasmic anti-viral mechanisms, and host or viral strategies that modulate the cell cycle to restrict or promote viral replication) could influence target cell susceptibility to viral infections. The imHC possess a higher density and variety of known surface DENV receptors (i.e., HSPGs and TIM-1) (**S2 Fig**), which likely results in a higher DENV binding capacity than Huh-7 and HepG2 (**Fig 1**). The expression of syndecan-1 and the role of heparan sulfate in DENV binding of imHC shown in this study correlate with the previous findings showing the function of heparan sulfate proteoglycans as a major DENV receptor in human hepatocytes [18,28]. However, the specific role of TIM-1 as a DENV receptor in hepatocytes has not yet been elucidated. In this study, we demonstrated that a reduction of plasma membrane TIM-1 expression on imHC by specific siRNA treatment did not affect cell susceptibility to DENV (**S3 Fig**). Indeed, the lack of detectable TIM-1 expression on the surface of primary hepatocytes (**S3A Fig**) confirmed the negligible role of TIM-1 for DENV entry into human hepatocytes. All of these findings suggest that TIM-1 is not a crucial DENV receptor on human hepatocytes.

Among the three hepatic cell types, imHC showed the highest efficiency in DENV replication and NS1 secretion than hepatoma cell lines, especially at the early time point with a low infection condition (**Fig 2**). These results suggest that imHC was more permissive to lower amounts of viral infection and had a faster rate of DENV replication and NS1 secretion than those of the hepatoma cell lines. However, unlike intracellular NS3 expression and NS1 secretion, no significant difference was observed for extracellular infectious virion production among the three cell types. This suggests that the hepatocytes might possess some underlying mechanisms for viral packaging and secretion that could compensate for the low percentages of viral infection. It is important to also note that levels of secreted NS1 correlated better with the number of infected cells than the amount of infectious virion released. This observation is in line with the previous study [66] showing that NS1 is likely secreted through a pathway independent of virion secretion mechanisms. Based on these findings, it is tempting to speculate that levels of secreted NS1 could serve as a better surrogate marker representing the magnitude of infected cell numbers (or virus burden within target cells) than virion production.

Interestingly, HepG2, which is the most commonly used cell type for *in vitro* DENV infection studies, showed the lowest efficiency in producing DENV-infected cells among the three cell types in this study. We also found that HepG2 grew faster (**S11 Fig**), yet underwent apoptotic death faster (50–60% of cell populations) than the other two cell lines following DENV infection (**Fig 4**). A previous report described multiple apoptotic pathways activated by DENV in HepG2 (3). DENV antigens in the infected cells might be degraded once the cells undergo apoptosis, likely explaining the lower percentage of DENV-NS3 positive HepG2 cells detected in the kinetic experiments.

The major characteristic event in dengue pathogenesis is a cytokine storm triggered by DENV infection [41]. Several cytokines found in the circulation of severe dengue patients have been shown to be associated with plasma leakage, including TNFα and IL-8 [67]. Since hepatocytes serve as the major target of DENV infection, they are suggested to contribute to severe dengue pathogenesis as cytokine-producing cells. However, studies of cytokines expressed by hepatocytes upon DENV infection were mainly performed at the gene level or used hepatoma cell lines as the cell model. Information on cytokine protein production from PHHs upon DENV infection remains scarce. In this study, we characterized DENV-induced cytokine expression of PHHs at both protein and gene levels in comparison with those of imHC and the hepatoma cell lines. Results revealed secretion of 9 cytokines (TNF-α, IL-8, MIP-1α, MIP-1β, MCP-1, RANTES, IL-1rα, IL-9 and IP-10) from primary hepatocytes (**Fig 5**). TNF-α, MIP-1β, IL-8 and IP-10, which have been described for their significant roles in dengue pathogenesis [68–71], were also produced by imHC, and the other two hepatic cell lines (except that TNF-α secretion was not detectable in Huh-7). Notably, IP-10 and IL-8 were secreted at relatively high levels (>200 pg/ml) by primary hepatocytes and three hepatic cell lines, suggesting preeminent roles of these cytokines in human hepatocytes. Unlike PHHs, imHC could not secrete detectable levels of MCP-1, MIP-1α, and RANTES although DENV-induced gene expression patterns of these cytokines were similar between imHC and PHHs (**Figs 5D–5F and S5**). The MCP-1 expression was not detectable in HepG2 and Huh-7 before and following viral challenge; the lack of MCP-1 gene expression in HepG2 was similar to a previous observation [72]. Notably, MCP-1 expression levels were significantly higher in DHF/DSS than in the DF patients [73,74], implicating involvement in dengue pathogenesis. Indeed, MCP-1 could directly perturb endothelial cell tight junction protein distribution, and contribute to increased vascular permeability [74]. Additionally, RT-PCR revealed a dose-dependent increase of TRAIL-γ, previously implicated as an anti-apoptotic factor [75], in imHC upon DENV infection but decreased levels of this cytokine transcript in HepG2 (**Fig 5J**). This likely explains, at least in part, the higher resistance to DENV-induced apoptosis that is more characteristic of imHC than HepG2.

Among the three cell lines, HepG2 was the most effective in terms of cytokine secretion. The majority of cytokines produced by PHHs could also be detected in HepG2. However, the secreted levels of several cytokines (i.e., TNF-α, IL-8 and RANTES) from HepG2 were much higher than those of PHHs following 48 hours of DENV infection. The unusually high cytokine levels of HepG2 could be a result of the extremely high rate of cell proliferation and DENV-induced apoptotic cell death, which significantly occurred in HepG2 but not in imHC (**Fig 4**). Of note, in the liver tissues of fatal DENV-infected cases, hepatocytes are massively infected and harbor large amounts of viral antigens and genomes indicating that the liver is a major viral-replicating site; however, a surprisingly low degree of apoptosis was detected in liver cells [42,76]. It has been described for DENV that host cell apoptosis was either inhibited or delayed by viral gene products especially at the early phase of infection to support viral growth [77]. Altogether, our results suggest that imHC efficiently supports DENV replication yet offers resistance to virus-induced apoptotic cell death even at conditions of high viral burden (resembling *in vivo* pathology). These unique characteristics make it suitable for use as an alternative hepatocyte model to study DENV infection *in vitro* despite some dissimilarity in the cytokine secretion profile as compared to PHHs.

Lipid droplets, heterogeneous in size and function, are now recognized as multifunctional organelles playing pivotal roles in cell biology and metabolism [78,79]. Others have shown that DENV infection triggers lipid droplet autophagy and the released free fatty acids are available for oxidation and ATP production; this lipophagy process facilitates DENV replication [47,80]. This phenomenon likely explains our results of decreased lipid droplet surface area in DENV-infected imHC and Huh-7 [47]. However, unlike the previous study [81], we observed no change in lipid droplet area within DENV-infected HepG2, which could be due to any of a number of factors including different culture conditions. Additionally, lipid droplets in imHC and Huh-7 were strikingly larger in size than those of HepG2, although the significance of this observation is unclear at this time. Of note though, Huh-7 was more responsive to exogenous fat supply than HepG2, thereby synthesizing and storing more TAG, the major constituent enriched in lipid droplets [82]. Moreover, unlike HepG2, hepatocyte-like cells derived from human induced pluripotent stem cells displayed similar profiles of key enzymes of lipid metabolism and fatty acids when compared to primary human hepatocytes [83].

Herein we further characterized the overall changes of lipid droplets along with TAG levels in response to DENV infection in hepatocytes using mass spectrometry. This provided more detailed information on the molecular species involved than thin layer chromatography which was employed previously [47]. Despite with 20-fold lower infection dose used in our experiments, a decrease in the levels of palmitic acid-containing TAG (C16:0-TAG) in imHC were more apparent than in the other two hepatoma cell lines. These findings agreed with the previous study showing a reduction of C16:0-TAG in DENV-infected mosquito cells. The reduction was caused by DENV-enhanced catabolism of C16:0-TAG to generate C16:0 fatty acids. This is a major building block for phospholipid biosynthesis, a process critical for DENV replication [84]. Our lipidomic profiling further revealed changes of specific species of C16:0-TAG (C48:0 and C50:0) during DENV infection in human hepatocytes. Of note, C16:0-TAG was also found to be the most abundant TAG species of PHHs (**S12 Fig**). Further studies to determine the roles of these C16:0-TAG species in the DENV life cycle in human hepatocytes are warranted to specifically identify potential strategies for intervention that could restrict viral growth. Notably, the alterations in cellular lipid profiles at a low DENV burden were exclusively observed in the imHC, suggesting somewhat more dynamic lipid metabolism in these cells. This is another distinct characteristic of this cell type.

In terms of drug testing, imHC showed remarkable responses to the two drugs with anti-viral activity tested in this study. The degrees of reduction in DENV replication, NS1 secretion and virion production of imHC in response to drug treatment were more pronounced than those of the hepatoma cell lines, especially HepG2. Supporting this notion, the expression of various drug-metabolizing enzymes of the CYP450 family in HepG2 appeared to be less readily inducible than in the other hepatic cell types [85]. Previous findings and our own results raise concern for continued use of HepG2 as an *in vitro* model for assessment of anti-viral drug efficacy and mechanism of actions in response to DENV. Interestingly, the inhibitory effects of ivermectin on DENV replication/production were more apparent than those of ribavirin, suggesting that these two drugs likely employ different mechanisms of action in hepatocytes. In addition to ribavirin and ivermectin, our recently published study demonstrated that imHC respond well to common HBV therapeutic treatments, including direct-acting antiviral drugs (DAAs) and interferons (IFNs) [65], emphasizing the suitability of imHC as a platform for multi anti-viral drug screening and testing.

While mouse models are a powerful tool for *in vivo* studies of the pathogenesis of liver disease and drug discovery, their cost and associated technical challenges make them impractical for early stage drug screening. Thus, a suitable *in vitro* model is needed to advance research in this area. Although imHC possess the Bmi-1 oncogene, which is absent from primary hepatocytes, its cell proliferation and apoptosis rates were not as aberrantly high as in the hepatoma cell lines. The superior efficiency of imHC in response to drug treatment and DENV infection at a low viral burden, which likely occurs at physiological conditions, makes this cell line very attractive as a model to study DENV *in vitro*. In conclusion, this study has provided compelling evidence for the use of imHC as a more appropriate alternative *in vitro* cellular model for initial high-throughput drug screening for therapeutic discovery as well as studying cellular responses in liver diseases, especially in hepatotropic infectious diseases like dengue.

## Supporting information

S1 Fig Immunolocalization of selected hepatocyte markers in imHC cells and the two commonly used hepatic cell lines. Cells were seeded in a 96-CellCarrier-96 Black plate (PerkinElmer) and maintained for three days. Cells were fixed with 4% paraformaldehyde in PBS, permeabilized using 0.1% Triton X-100 in PBS, and then blocked with 3% (w/v) BSA in PBS for 30 min at 37°C. Primary antibodies against hepatic markers included anti-albumin/ALB (1:500 dilution, AB10241, Abcam), anti-α-fetoprotein/AFP (1:100 dilution, SC8399, Santa Cruz Biotechnology), anti-low-density lipoprotein receptor/LDLR (1:100 dilution, SC373830, Santa Cruz Biotechnology), anti-multidrug resistance-associated protein 2/MRP2 (1:100 dilution, AB3373, Abcam), and anti-hepatocyte nuclear factor 4α/HNF-4α (1:100, SC6556, Santa Cruz Biotechnology). IgG isotypes corresponding to the primary antibodies were included as negative controls. Following the primary antibody incubation (37°C, 1 h), the cells were washed thrice with PBS and incubated with the corresponding fluorophore-conjugated secondary antibodies (37°C, 40 min). The host nuclei were stained with Hoechst 33342. Fluorescent imaging was performed using Operetta High-Content Imaging System (PerkinElmer) at 40x magnification. Arrows indicate the bile canalicular-like structure of MRP staining, previously reported in HepG2 cells. [86] Scale bar = 10 μm.
(TIF)

S2 Fig Expression of DENV receptors in hepatic cell lines and PHHs. Equal amounts of total protein (20 μg) extracted from each cell type were resolved on 10% SDS polyacrylamide gels and subjected to Western blotting analysis for specific DENV receptors, including syndecan-1 (**A**), TIM-1 (**B**), TIM-4 (**C**), AXL (**D**), TYRO3 (**E**). Proteins extracted from HEK-TIM1, HEK-TIM4, Vero and U87-MG cells were included in the experiments as positive controls for specific DENV receptor expression. The surface expression of TIM-1, TIM-4 and AXL on HEK-TIM1 (**F**), HEK-TIM4 (**G**), and Vero cells (**H**), respectively was also confirmed by flow cytometry. Isotype control was used in place of primary antibody as a negative control for both Western blotting and flow cytometry. GAPDH was used an endogenous protein control for immunoblotting.
(TIF)

S3 Fig Confirmation of TIM-1 expression in hepatocytes. (**A**) Representative images from confocal microscopic analysis showing TIM-1 expression on the surface of imHC and Huh-7 cells but not on HepG2 cells and primary human hepatocyte. To verify the role of TIM-1 in imHC cells, the imHC cells were transiently transfected (24 h) by siRNA targeting TIM-1 (SiTIM1) or nontargeting siRNA (SiCon) using Lipofectamine RNAiMax (Life Technologies Inc.) following the manufacturer’s protocol. After siRNA transfection, the cells were subjected to immunofluorescent staining and flow cytometry. (**B**) The flow cytometry histograms showing decreased TIM-1 expression levels in SiTIM1 cells (blue line) as compared to the TIM-1 level of SiCon cells (orange line). Goat IgG (green line) was used as an isotype control to show no background (non-specific) staining of the secondary antibody. The SiTIM1 and SiCon cells were further infected with DENV-2 at MOI of 0.5 for 24 h, and then the cells were stained for intracellular NS3 to determine the DENV infection levels by flow cytometry (**C**) and the culture supernatants of infected cells were subjected to FFU assay (**D**). Results show no significant reduction of DENV replication efficiency in imHC cells following the siRNA knockdown of TIM-1. Data in (**D**) are mean + S.D. of values from three experiments.
(TIF)

S4 Fig Morphology and DENV production of PHHs from 2 donors. The PHHs from 2 individuals were obtained from Thermo Scientific Inc. and cultured under conditions according to the company’s instructions. (**A**) Representative light microscopic images of primary hepatocytes of the 2 donors after culturing in serum-free cell incubation medium overnight prior to DENV-2 infection. Scale bar = 200 μM. The primary hepatocytes were infected with DENV-2 (MOI of 5) for 48 h and the culture supernatants were collected for determination of viral RNA (**B**) and viral production (**C**) by qRT-PCR and FFU assay, respectively. Plots show the average of data from duplicates of each donor.
(TIF)

S5 Fig Cytokine gene expression in hepatocytes. The three hepatic cell lines and PHHs from 2 different donors (D1 and D2) were mock-infected (M) or infected with DENV-2 (DV) at MOI of 5. At the specified time points, cells were harvested for assessment of cytokine gene expression. An equal number of each cell type was used for RNA extraction and an equal amount of RNA from each source was further subjected to RT-PCR and gel electrophoresis. Representative gel images of RT-PCR products corresponding to the quantified cytokine proteins in Fig 5 are shown in panels **A-F**. The 18S rRNA was used as a loading control (**G**).
(TIF)

S6 Fig Representative chromatograms and MS/MS spectra from neutral loss scanning of 273 showing profiles of TAG with C16:0 in three hepatic cell lines, PHHs, PBMCs, and U87-MG glioma cell line. (**A**) Representative total ion chromatograms of lipid extracts from different cell types from 80-min gradient separation on the reversed-phase-UPLC system and the neutral loss scanning of 273 for precursors containing a C16:0 fatty acyl chain. The peaks corresponding to TAG species were found in the 45–60 min part of the gradient. The total ion profiles obtained from the neutral loss scans of imHC, Huh-7 and HepG2 cell lipid extracts were similar to that of PHH but different from those of non-hepatic cell types (PBMCs, U87-MG). (**B**) Representative MS/MS spectra from the neutral loss scanning of 273 of ammoniated TAGs in lipid extracts from different cell types. The major *m/z* signals (as indicated with bold numbers) obtained from this neutral loss scan were similar among the hepatic cell types but different from those of the non-hepatic cells.
(TIF)

S7 Fig Representative chromatograms and MS/MS spectra from neutral loss scanning of 301 showing profiles of TAG with C18:0 in three hepatic cell lines, PHHs, PBMCs, and U87-MG glioma cell line. (**A**) Representative total ion chromatograms from neutral loss scanning of 301 for precursors containing a C18:0 fatty acyl chain also showed similar ion profiles in 4 hepatic cell types, but different from those of the PBMCs and U87-MG. (**B**) Representative MS/MS spectra from the neutral loss scanning of 301 showing similar relative intensity of major *m/z* signals (as indicated with bold numbers) among the 4 hepatic cell types.
(TIF)

S8 Fig No changes of dipalmitoylphosphatidylcholine (DPPC) following DENV infection in the three hepatic cell lines. The abundances of DPPC or PC with C16:0/16:0 (*m/z* 734), a major phospholipid that exists in hepatocytes, were determined based on the peak areas of the extracted ion chromatograms of DPPC (m/z 734) from precursor ion scanning of m/z 184 in three hepatic cell line samples. Statistical analysis (Student’s *t*-test) shows no significant differences (ns) between DPPC levels of MOCK and DENV-infected cells of each cell type.
(TIF)

S9 Fig Structures and fragmentation patterns of TAG with C48:0 and C50:0. The MS/MS fragmentation of TAG generates losses of a neutral palmitic acyl chain (C_16_H_32_O_2_+NH_3_) with a mass of 273 from the *sn* positions of glycerol backbone (indicated by dotted lines) and this neutral mass loss can be detected by the neutral loss scanning modality of MS/MS analysis.
(TIF)

S10 Fig Anti-viral effects of ivermectin on PHHs. The PHHs from 2 donors were infected with DENV-2 at MOI of 0.1 and cultured under the following conditions: the complete medium without any treatment (Control), the complete medium containing 0.5% DMSO (DMSO), or the complete medium containing 5 μM of ivermectin solubilized in 0.5% DMSO (Ivermectin). Following 48 h of culture, the cells and culture supernatants were collected for assessment of ivermectin effects on viral production based on the RNA levels quantified by RT-PCR (**A**) or infectious viral particle levels determined by FFU assay (**B**). Data are presented as mean ± S.D. of values from triplicates of each donor. The asterisk indicated a statistically significant difference (*P* < 0.05) between DMSO and ivermectin conditions.
(TIF)

S11 Fig Comparison of cell proliferation of three hepatic cell lines during 48 hours of cell culture. The three cell lines (HepG2, Huh-7, imHC) were plated in 24-well plates (2 x 10^5^ cells/well) and cultured in their corresponding media overnight. The cells were mock infected or infected with DENV-2 at MOI of 5. At 0, 6, 12, 24 and 48 h of cell culture, the cells were detached from the wells by incubation in 0.1% trypsin in 2.5 mM EDTA in PBS (3 min, 37°C), and subjected to cell counting using a hemocytometer under a Zeiss inverted microscope. The numbers of cells per well of the 24-well plate were plotted as a function of time. The data are presented as mean ± S.D. of values from three independent experiments. Asterisks indicate significant differences between HepG2 and Huh-7 cells (*P* = 0.0077) and HepG2 and imHC cells (*P* = 0.0458) at 48 h of cell culture.
(TIF)

S12 Fig Proportion of 4 major TAG species in hepatic cell lines and PHHs. The relative abundances of major TAG species, containing different fatty acyl chains (C16:0, C16:1, C18:0 and C18:1) in different hepatic cell types were obtained from MS/MS analysis using neutral loss scanning modes as described in the Methods. The proportions of each TAG species to the sum amounts of all species in different cell types were shown in the graph.
(TIF)

S1 Table List of major m/z signals detected in the four neutral loss scans of hepatocyte lipid extracts and their possible identities as TAG species. (PDF)

S2 Table CC50 and EC50 values of ivermectin and ribavirin. (PDF)

S3 Table Viability of hepatic cell lines after DENV infection and drug treatment. (PDF)
