# Supplementary figures and images for: Immortalized stem cell-derived hepatocyte-like cells: An alternative model for studying dengue pathogenesis and therapy
Source: PLoS Negl Trop Dis. 2020 Nov 20;14(11):e0008835. doi: 10.1371/journal.pntd.0008835 (PMC7717553; doi:10.1371/journal.pntd.0008835)

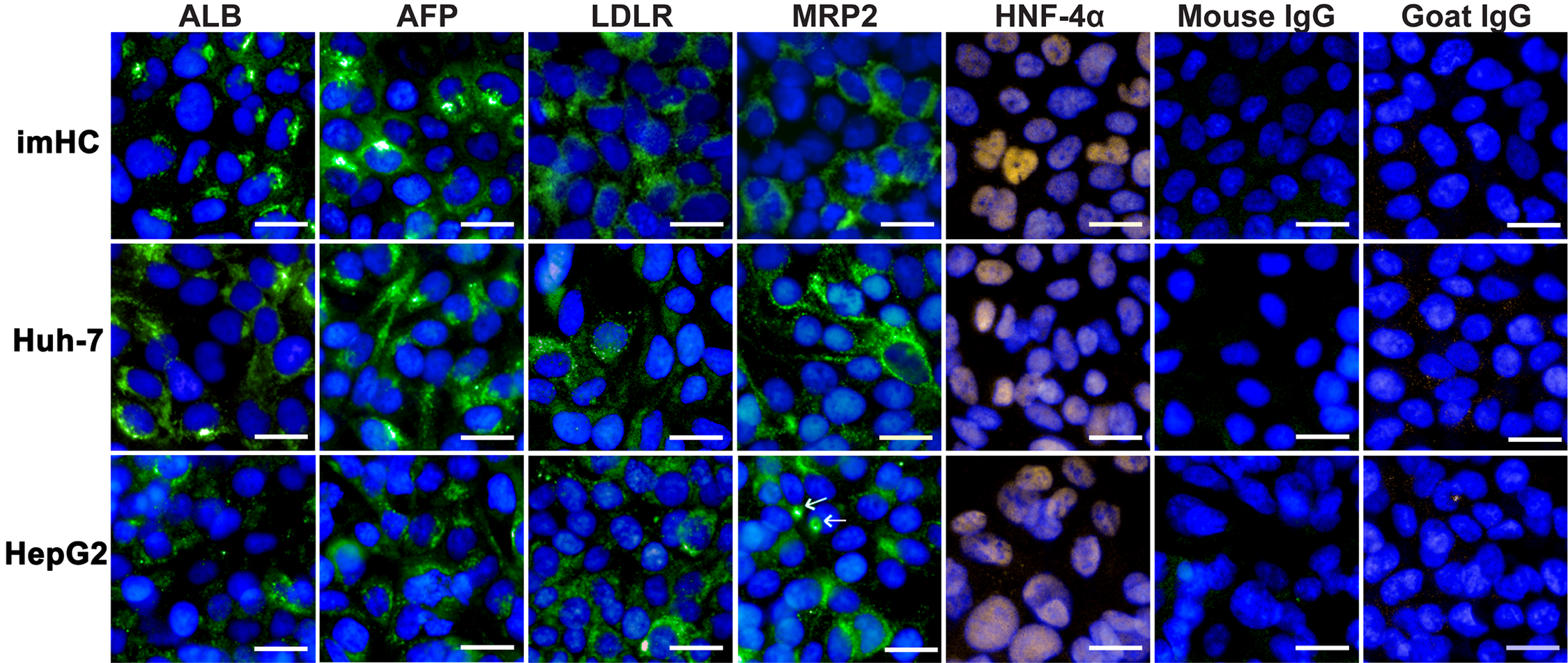

Supplement: S1 Fig — Cells were seeded in a 96-CellCarrier-96 Black plate (PerkinElmer) and maintained for three days. Cells were fixed with 4% paraformaldehyde in PBS, permeabilized using 0.1% Triton X-100 in PBS, and then blocked with 3% (w/v) BSA in PBS for 30 min at 37°C. Primary antibodies against hepatic markers included anti-albumin/ALB (1:500 dilution, AB10241, Abcam), anti-α-fetoprotein/AFP (1:100 dilution, SC8399, Santa Cruz Biotechnology), anti-low-density lipoprotein receptor/LDLR (1:100 dilution, SC373830, Santa Cruz Biotechnology), anti-multidrug resistance-associated protein 2/MRP2 (1:100 dilution, AB3373, Abcam), and anti-hepatocyte nuclear factor 4α/HNF-4α (1:100, SC6556, Santa Cruz Biotechnology). IgG isotypes corresponding to the primary antibodies were included as negative controls. Following the primary antibody incubation (37°C, 1 h), the cells were washed thrice with PBS and incubated with the corresponding fluorophore-conjugated secondary antibodies (37°C, 40 min). The host nuclei were stained with Hoechst 33342. Fluorescent imaging was performed using Operetta High-Content Imaging System (PerkinElmer) at 40x magnification. Arrows indicate the bile canalicular-like structure of MRP staining, previously reported in HepG2 cells. [86] Scale bar = 10 μm. (TIF) [file pntd.0008835.s001.tif]

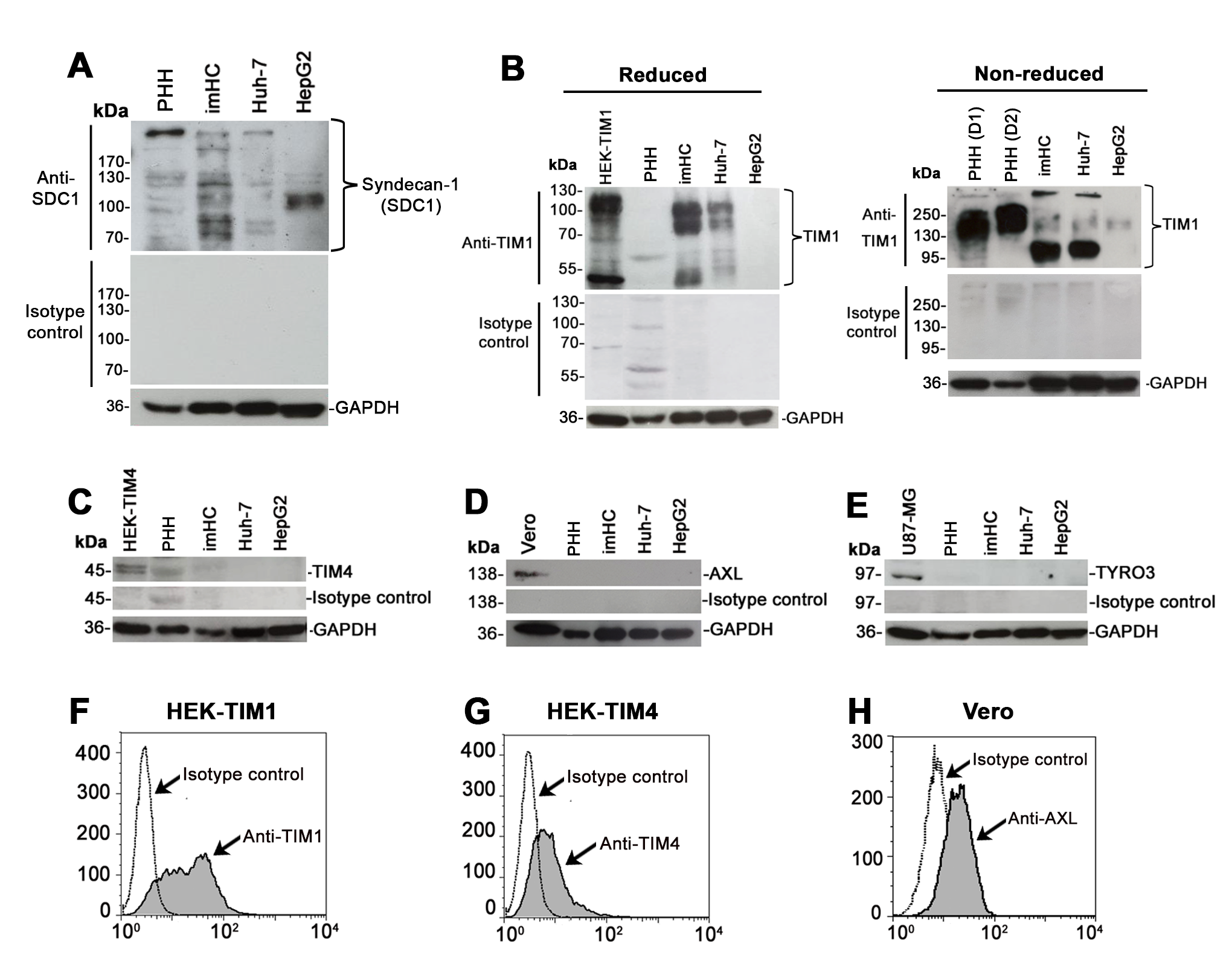

Supplement: S2 Fig — Equal amounts of total protein (20 μg) extracted from each cell type were resolved on 10% SDS polyacrylamide gels and subjected to Western blotting analysis for specific DENV receptors, including syndecan-1 (A), TIM-1 (B), TIM-4 (C), AXL (D), TYRO3 (E). Proteins extracted from HEK-TIM1, HEK-TIM4, Vero and U87-MG cells were included in the experiments as positive controls for specific DENV receptor expression. The surface expression of TIM-1, TIM-4 and AXL on HEK-TIM1 (F), HEK-TIM4 (G), and Vero cells (H), respectively was also confirmed by flow cytometry. Isotype control was used in place of primary antibody as a negative control for both Western blotting and flow cytometry. GAPDH was used an endogenous protein control for immunoblotting. (TIF) [file pntd.0008835.s002.tif]

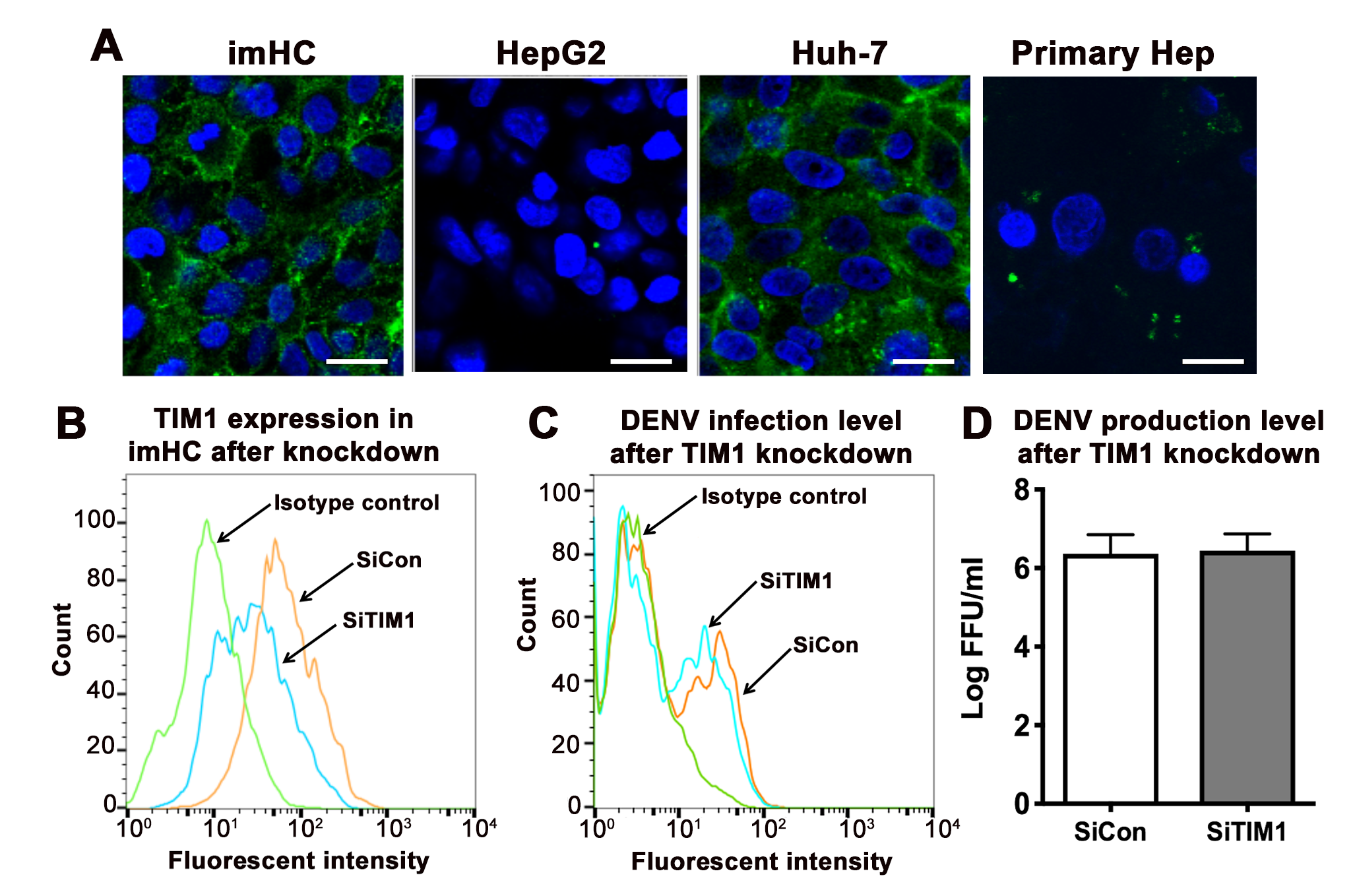

Supplement: S3 Fig — (A) Representative images from confocal microscopic analysis showing TIM-1 expression on the surface of imHC and Huh-7 cells but not on HepG2 cells and primary human hepatocyte. To verify the role of TIM-1 in imHC cells, the imHC cells were transiently transfected (24 h) by siRNA targeting TIM-1 (SiTIM1) or nontargeting siRNA (SiCon) using Lipofectamine RNAiMax (Life Technologies Inc.) following the manufacturer’s protocol. After siRNA transfection, the cells were subjected to immunofluorescent staining and flow cytometry. (B) The flow cytometry histograms showing decreased TIM-1 expression levels in SiTIM1 cells (blue line) as compared to the TIM-1 level of SiCon cells (orange line). Goat IgG (green line) was used as an isotype control to show no background (non-specific) staining of the secondary antibody. The SiTIM1 and SiCon cells were further infected with DENV-2 at MOI of 0.5 for 24 h, and then the cells were stained for intracellular NS3 to determine the DENV infection levels by flow cytometry (C) and the culture supernatants of infected cells were subjected to FFU assay (D). Results show no significant reduction of DENV replication efficiency in imHC cells following the siRNA knockdown of TIM-1. Data in (D) are mean + S.D. of values from three experiments. (TIF) [file pntd.0008835.s003.tif]

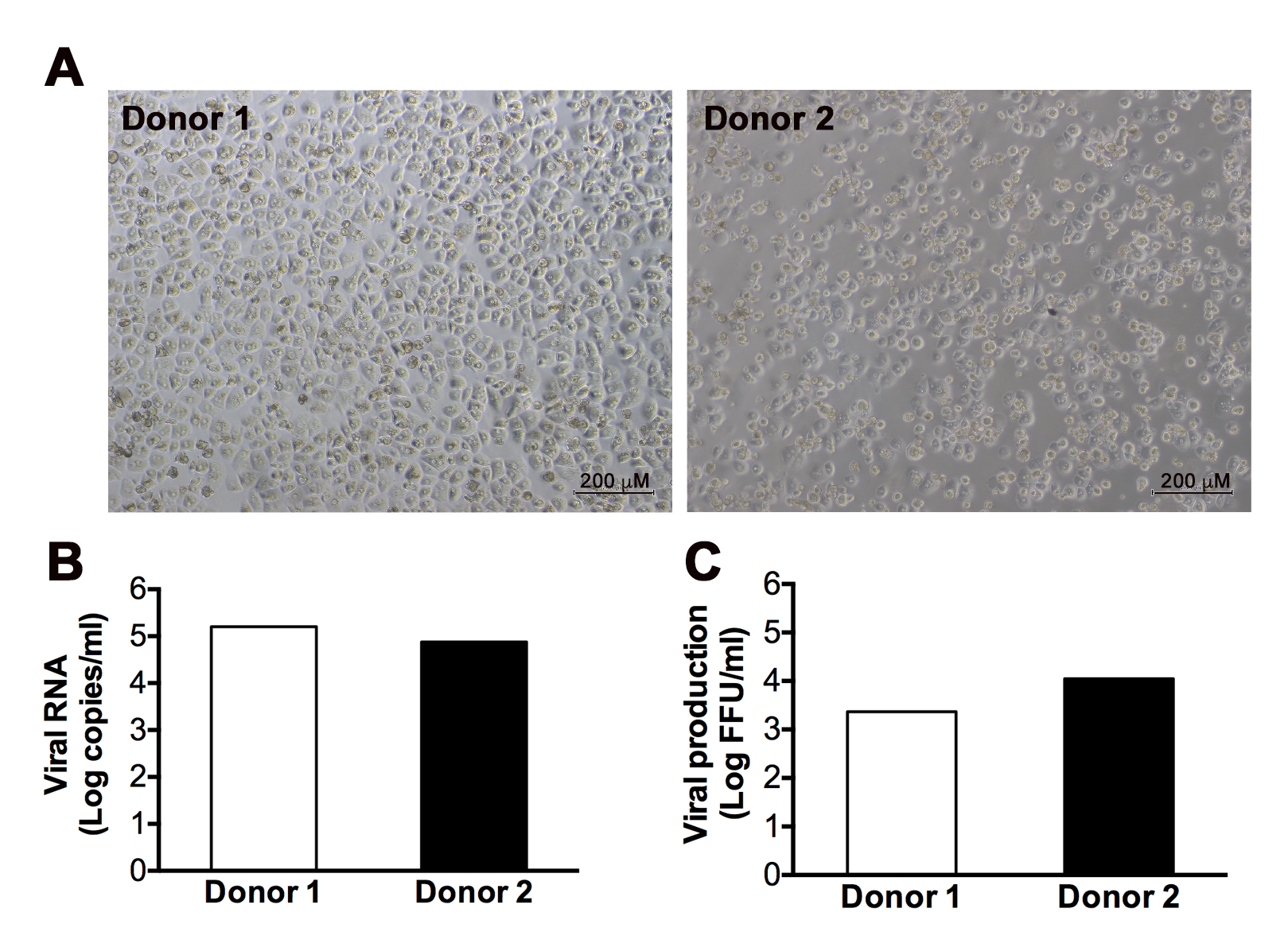

Supplement: S4 Fig — The PHHs from 2 individuals were obtained from Thermo Scientific Inc. and cultured under conditions according to the company’s instructions. (A) Representative light microscopic images of primary hepatocytes of the 2 donors after culturing in serum-free cell incubation medium overnight prior to DENV-2 infection. Scale bar = 200 μM. The primary hepatocytes were infected with DENV-2 (MOI of 5) for 48 h and the culture supernatants were collected for determination of viral RNA (B) and viral production (C) by qRT-PCR and FFU assay, respectively. Plots show the average of data from duplicates of each donor. (TIF) [file pntd.0008835.s004.tif]

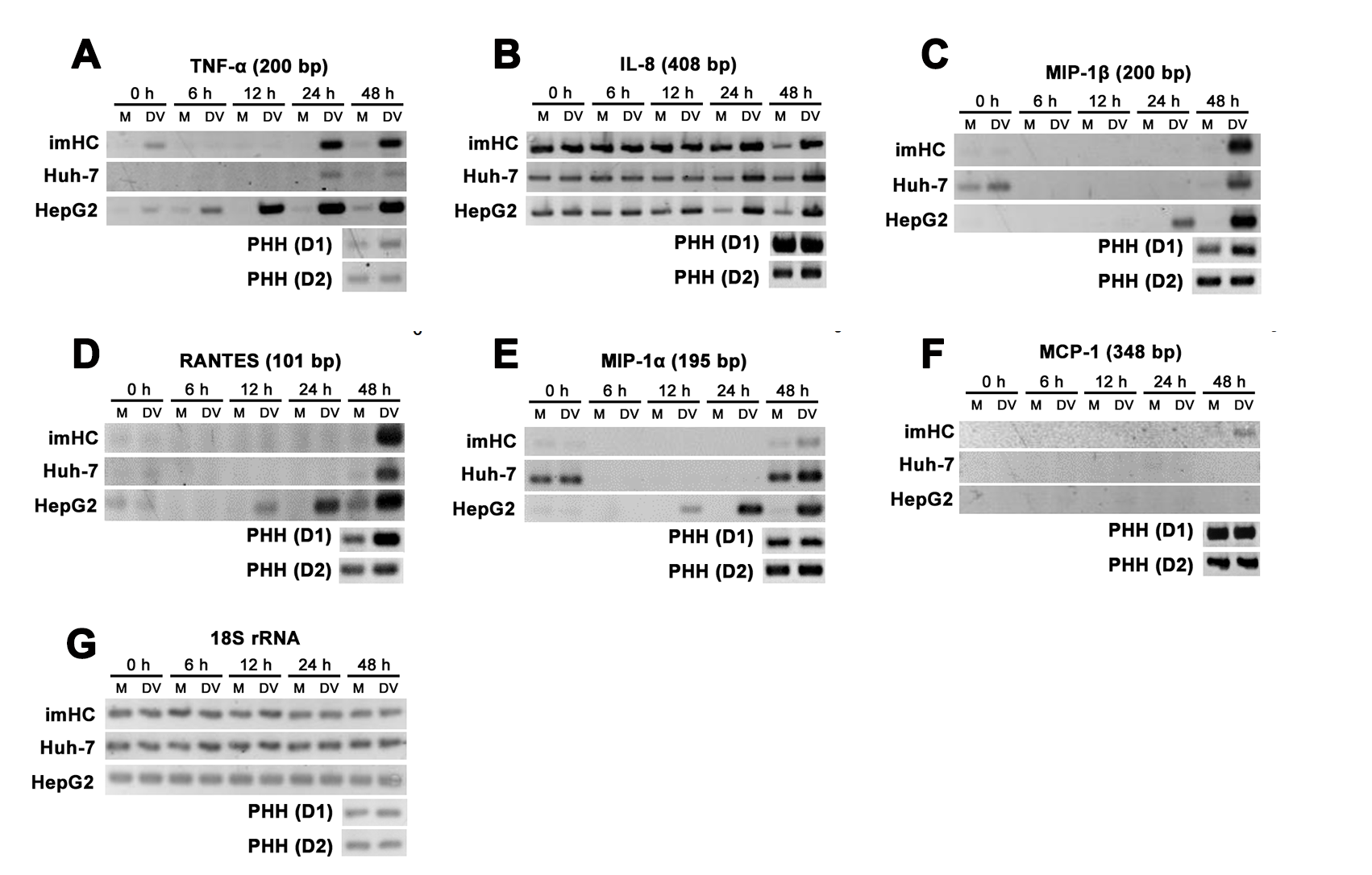

Supplement: S5 Fig — The three hepatic cell lines and PHHs from 2 different donors (D1 and D2) were mock-infected (M) or infected with DENV-2 (DV) at MOI of 5. At the specified time points, cells were harvested for assessment of cytokine gene expression. An equal number of each cell type was used for RNA extraction and an equal amount of RNA from each source was further subjected to RT-PCR and gel electrophoresis. Representative gel images of RT-PCR products corresponding to the quantified cytokine proteins in Fig 5 are shown in panels A-F. The 18S rRNA was used as a loading control (G). (TIF) [file pntd.0008835.s005.tif]

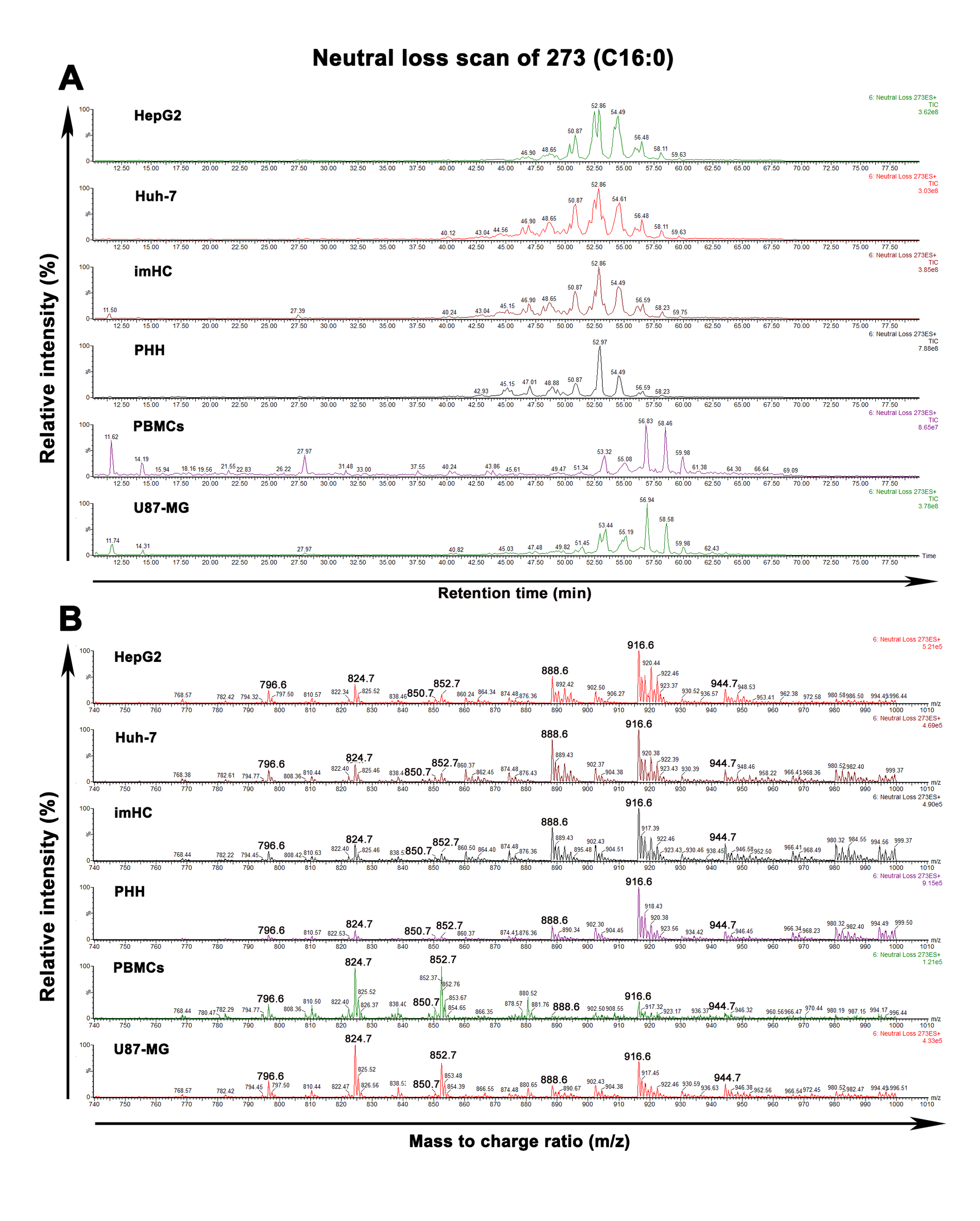

Supplement: S6 Fig — (A) Representative total ion chromatograms of lipid extracts from different cell types from 80-min gradient separation on the reversed-phase-UPLC system and the neutral loss scanning of 273 for precursors containing a C16:0 fatty acyl chain. The peaks corresponding to TAG species were found in the 45–60 min part of the gradient. The total ion profiles obtained from the neutral loss scans of imHC, Huh-7 and HepG2 cell lipid extracts were similar to that of PHH but different from those of non-hepatic cell types (PBMCs, U87-MG). (B) Representative MS/MS spectra from the neutral loss scanning of 273 of ammoniated TAGs in lipid extracts from different cell types. The major m/z signals (as indicated with bold numbers) obtained from this neutral loss scan were similar among the hepatic cell types but different from those of the non-hepatic cells. (TIF) [file pntd.0008835.s006.tif]

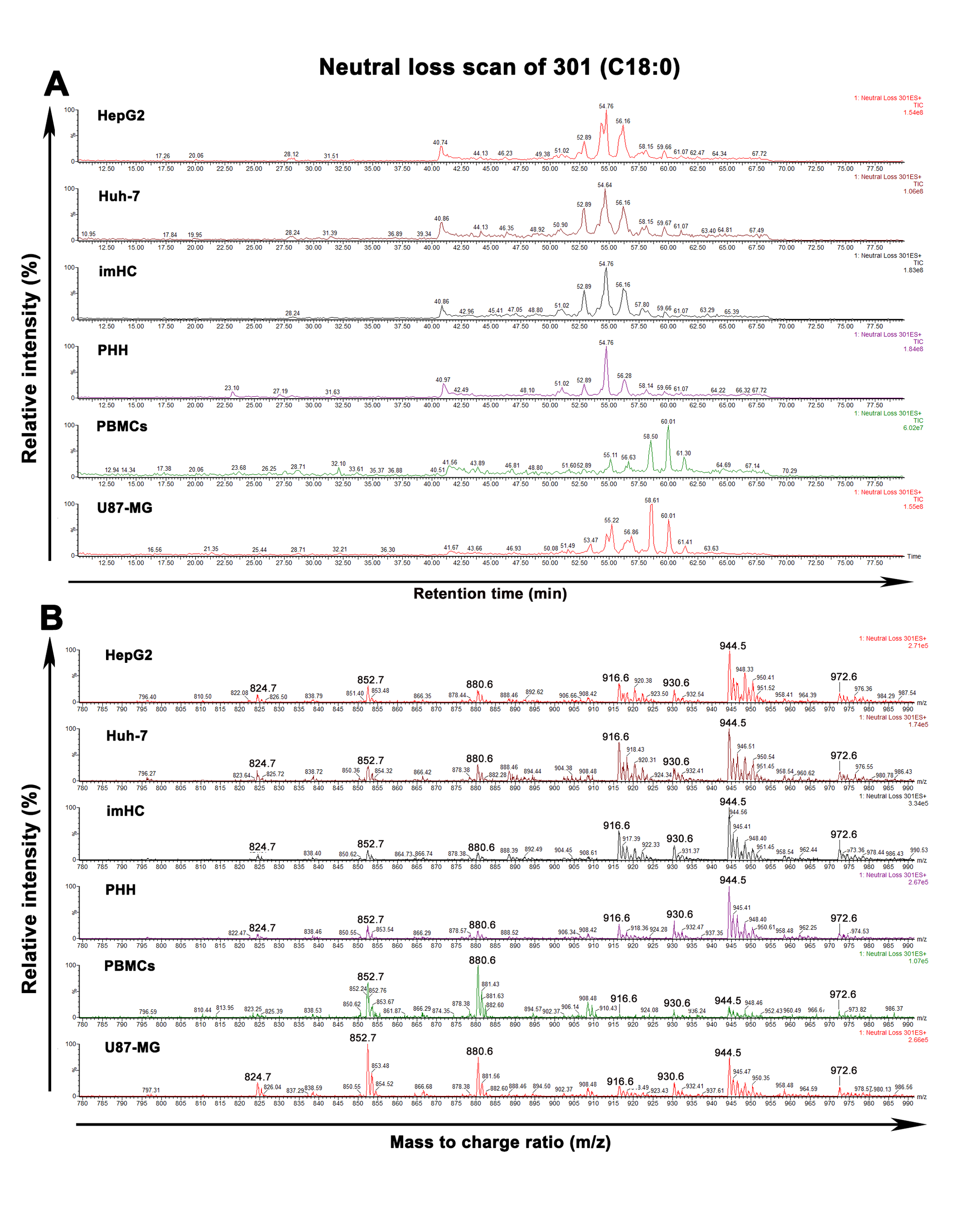

Supplement: S7 Fig — (A) Representative total ion chromatograms from neutral loss scanning of 301 for precursors containing a C18:0 fatty acyl chain also showed similar ion profiles in 4 hepatic cell types, but different from those of the PBMCs and U87-MG. (B) Representative MS/MS spectra from the neutral loss scanning of 301 showing similar relative intensity of major m/z signals (as indicated with bold numbers) among the 4 hepatic cell types. (TIF) [file pntd.0008835.s007.tif]

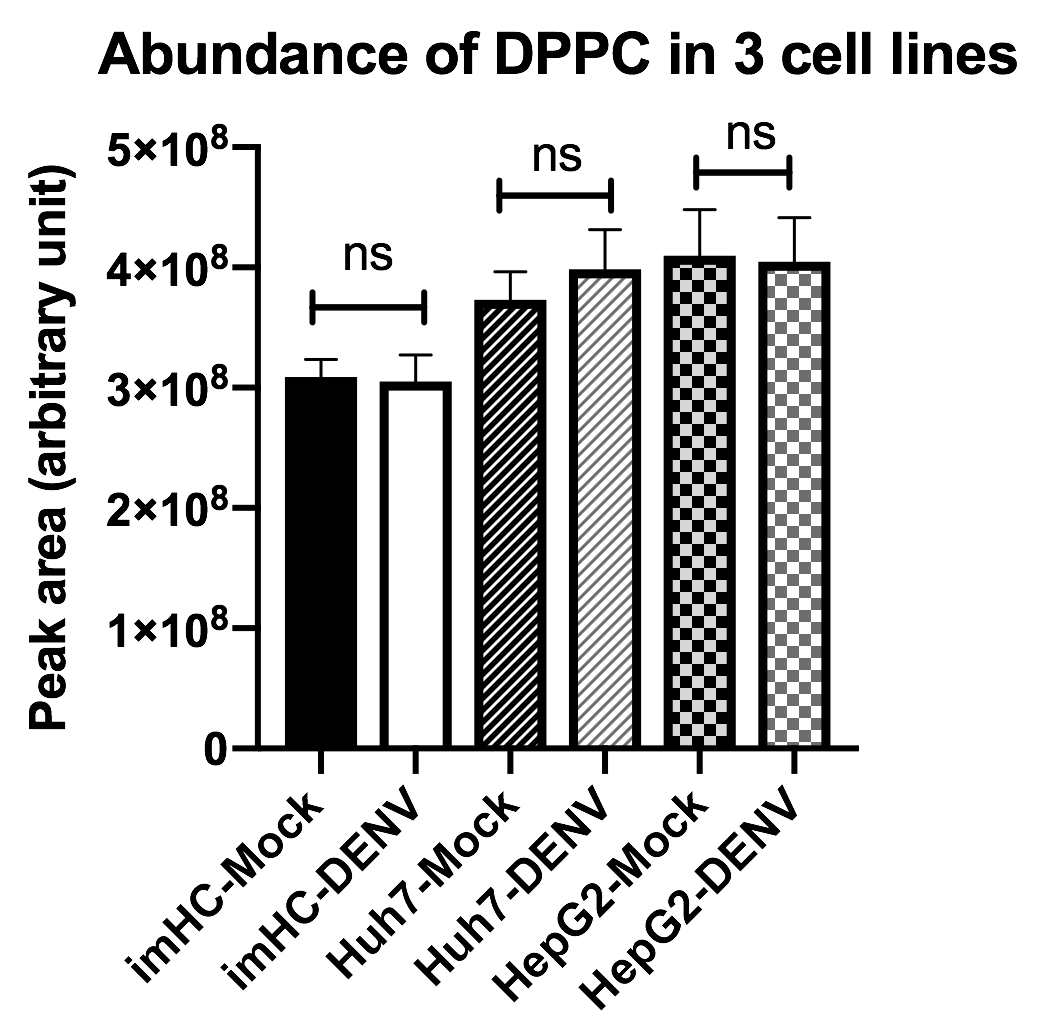

Supplement: S8 Fig — The abundances of DPPC or PC with C16:0/16:0 (m/z 734), a major phospholipid that exists in hepatocytes, were determined based on the peak areas of the extracted ion chromatograms of DPPC (m/z 734) from precursor ion scanning of m/z 184 in three hepatic cell line samples. Statistical analysis (Student’s t-test) shows no significant differences (ns) between DPPC levels of MOCK and DENV-infected cells of each cell type. (TIF) [file pntd.0008835.s008.tif]

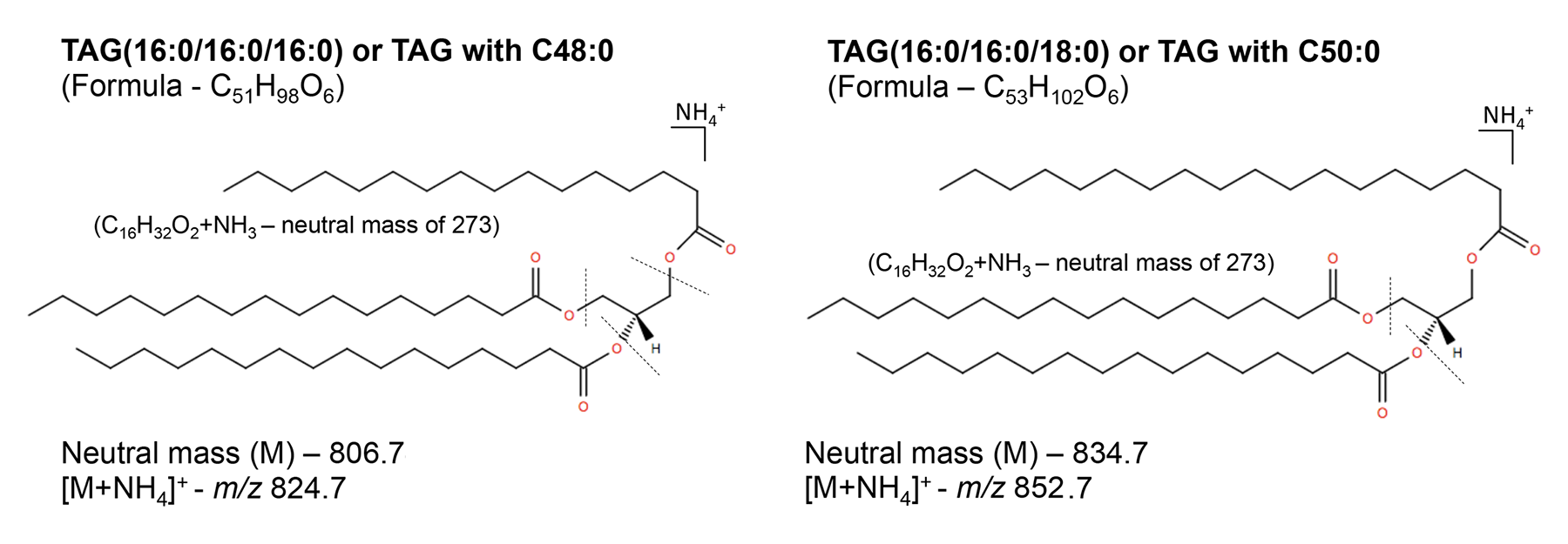

Supplement: S9 Fig — The MS/MS fragmentation of TAG generates losses of a neutral palmitic acyl chain (C16H32O2+NH3) with a mass of 273 from the sn positions of glycerol backbone (indicated by dotted lines) and this neutral mass loss can be detected by the neutral loss scanning modality of MS/MS analysis. (TIF) [file pntd.0008835.s009.tif]

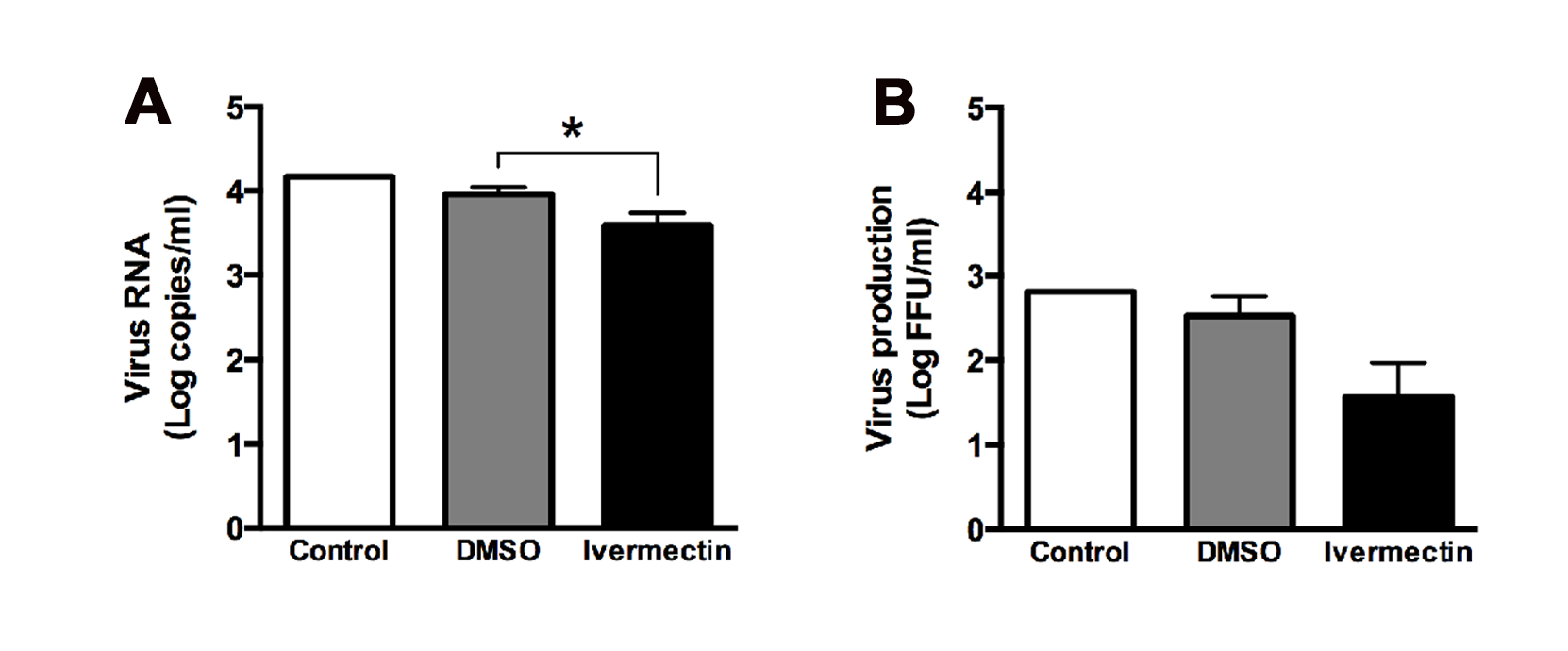

Supplement: S10 Fig — The PHHs from 2 donors were infected with DENV-2 at MOI of 0.1 and cultured under the following conditions: the complete medium without any treatment (Control), the complete medium containing 0.5% DMSO (DMSO), or the complete medium containing 5 μM of ivermectin solubilized in 0.5% DMSO (Ivermectin). Following 48 h of culture, the cells and culture supernatants were collected for assessment of ivermectin effects on viral production based on the RNA levels quantified by RT-PCR (A) or infectious viral particle levels determined by FFU assay (B). Data are presented as mean ± S.D. of values from triplicates of each donor. The asterisk indicated a statistically significant difference (P < 0.05) between DMSO and ivermectin conditions. (TIF) [file pntd.0008835.s010.tif]

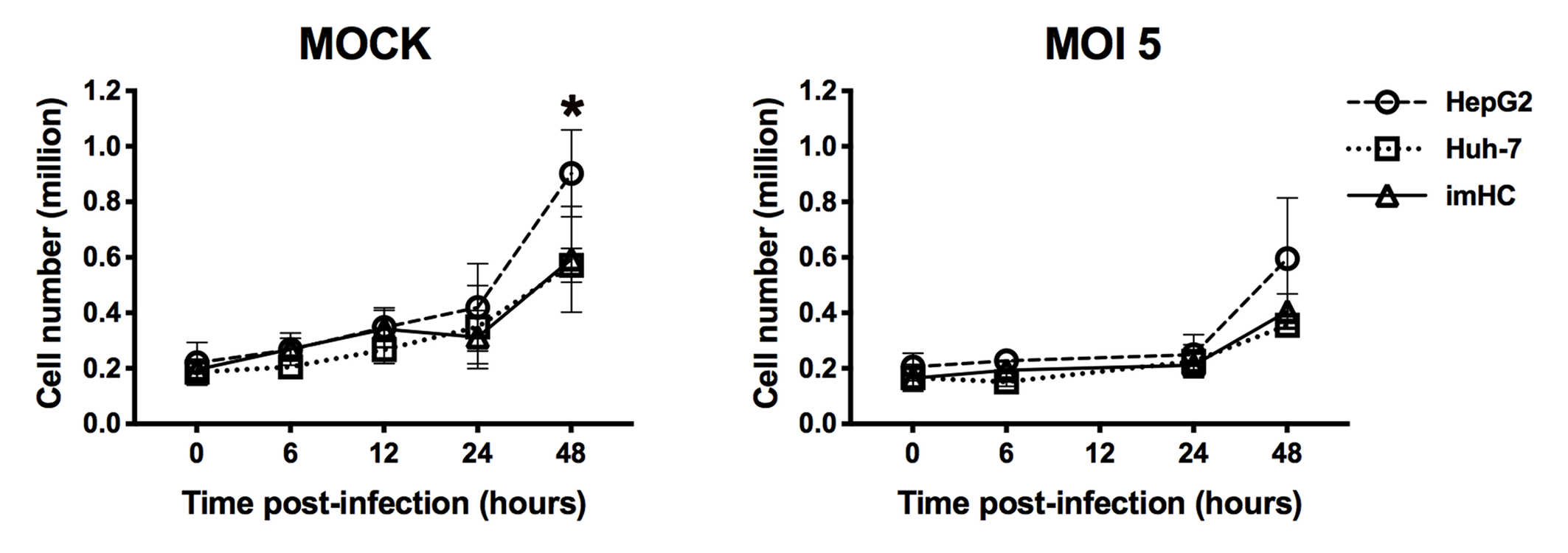

Supplement: S11 Fig — The three cell lines (HepG2, Huh-7, imHC) were plated in 24-well plates (2 x 10^5^ cells/well) and cultured in their corresponding media overnight. The cells were mock infected or infected with DENV-2 at MOI of 5. At 0, 6, 12, 24 and 48 h of cell culture, the cells were detached from the wells by incubation in 0.1% trypsin in 2.5 mM EDTA in PBS (3 min, 37°C), and subjected to cell counting using a hemocytometer under a Zeiss inverted microscope. The numbers of cells per well of the 24-well plate were plotted as a function of time. The data are presented as mean ± S.D. of values from three independent experiments. Asterisks indicate significant differences between HepG2 and Huh-7 cells (P = 0.0077) and HepG2 and imHC cells (P = 0.0458) at 48 h of cell culture. (TIF) [file pntd.0008835.s011.tif]

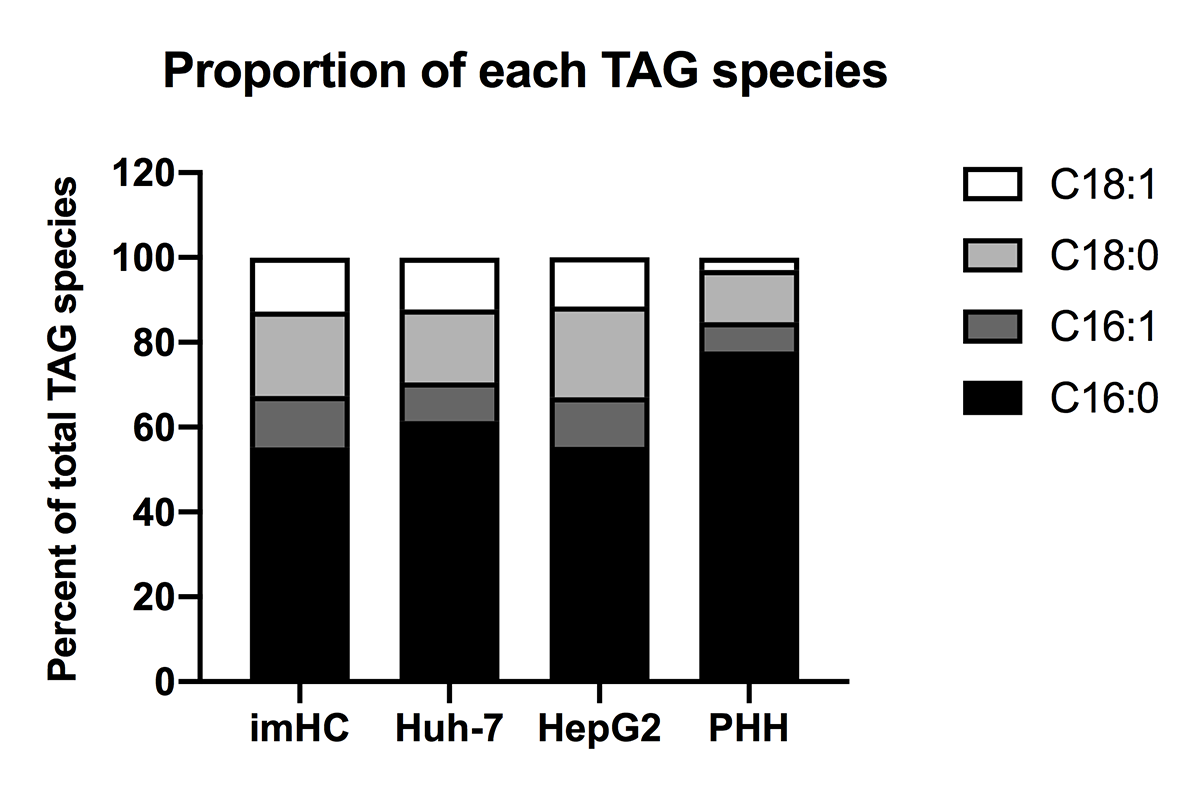

Supplement: S12 Fig — The relative abundances of major TAG species, containing different fatty acyl chains (C16:0, C16:1, C18:0 and C18:1) in different hepatic cell types were obtained from MS/MS analysis using neutral loss scanning modes as described in the Methods. The proportions of each TAG species to the sum amounts of all species in different cell types were shown in the graph. (TIF) [file pntd.0008835.s012.tif]
