# Supplementary material for: Immortalized stem cell-derived hepatocyte-like cells: An alternative model for studying dengue pathogenesis and therapy
Source: PLoS Negl Trop Dis. 2020 Nov 20;14(11):e0008835. doi: 10.1371/journal.pntd.0008835 (PMC7717553; doi:10.1371/journal.pntd.0008835)
Supplement: S1 Table — (PDF) [file pntd.0008835.s013.pdf]

S1 Table. List of major m/z signals detected in the four neutral loss scans of hepatocyte lipid extracts and their possible identities as TAG species

Neutral loss scan of	Found m/z	Formula $[M+NH_4]^+$	Total carbon and double bond numbers of fatty acyl chains	Examples of possible Acyl combination
**C16:0 (273)**	796.6	$C_{49}H_{94}O_6$+NH_4	C46:0	TAG(15:0/15:0/16:0)
	824.7	$C_{51}H_{98}O_6$+NH_4	C48:0	TAG(16:0/16:0/16:0)
	850.7	$C_{53}H_{100}O_6$+NH_4	C50:1	TAG(16:0/16:1/18:0)
	852.7	$C_{53}H_{102}O_6$+NH_4	C50:0	TAG(16:0/16:0/18:0)
	888.6	$C_{56}H_{102}O_6$+NH_4	C53:3	TAG(16:0/17:0/20:3)
	916.6	$C_{59}H_{94}O_6$+NH_4	C56:10	TAG(16:0/20:5/20:5)
	944.7	$C_{61}H_{98}O_6$+NH_4	C58:10	TAG(16:0/20:5/22:5)
**C18:0 (301)**	824.7	$C_{51}H_{98}O_6$+NH_4	C48:0	TAG(15:0/15:0/18:0)
	852.7	$C_{53}H_{102}O_6$+NH_4	C50:0	TAG(16:0/16:0/18:0)
	880.6	$C_{55}H_{106}O_6$+NH_4	C52:0	TAG(16:0/18:0/18:0)
	916.6	$C_{58}H_{106}O_6$+NH_4	C55:3	TAG(17:0/18:0/20:3)
	930.6	$C_{59}H_{108}O_6$+NH_4	C56:3	TAG(18:0/18:0/20:3)
	944.5	$C_{61}H_{98}O_6$+NH_4	C58:10	TAG(18:0/20:5/20:5)
	972.6	$C_{63}H_{102}O_6$+NH_4	C60:10	TAG(18:0/20:5/22:5)
**C16:1 (271)**	794.8	$C_{49}H_{92}O_6$+NH_4	C46:1	TAG(15:0/15:0/16:1)
	820.6	$C_{51}H_{94}O_6$+NH_4	C48:2	TAG(16:0/16:1/16:1)
	822.6	$C_{51}H_{96}O_6$+NH_4	C48:1	TAG(16:0/16:0/16:1)
	836.6	$C_{52}H_{98}O_6$+NH_4	C49:1	TAG(15:0/16:1/18:0)
	848.6	$C_{53}H_{98}O_6$+NH_4	C50:2	TAG(16:1/16:1/18:0)
	874.7	$C_{55}H_{100}O_6$+NH_4	C52:3	TAG(16:1/18:1/18:1)
	914.7	$C_{59}H_{92}O_6$+NH_4	C56:11	TAG(16:1/20:5/20:5)
	942.7	$C_{61}H_{96}O_6$+NH_4	C58:11	TAG(16:1/20:5/22:5)
**C18:1 (299)**	822.6	$C_{51}H_{96}O_6$+NH_4	C48:1	TAG(12:0/18:0/18:1)
	834.5	$C_{52}H_{96}O_6$+NH_4	C49:2	TAG(15:0/16:1/18:1)
	848.5	$C_{53}H_{98}O_6$+NH_4	C50:2	TAG(16:0/16:1/18:1)
	850.8	$C_{53}H_{100}O_6$+NH_4	C50:1	TAG(16:0/16:1/18:0)
	862.6	$C_{54}H_{100}O_6$+NH_4	C51:2	TAG(16:1/17:0/18:1)
	876.7	$C_{55}H_{102}O_6$+NH_4	C52:2	TAG(16:0/18:1/18:1)
	902.5	$C_{57}H_{104}O_6$+NH_4	C54:3	TAG(18:1/18:1/18:1)
	904.8	$C_{57}H_{106}O_6$+NH_4	C54:2	TAG(18:0/18:1/18:1)
	914.8	$C_{58}H_{104}O_6$+NH_4	C55:4	TAG(15:0/18:1/22:3)
	942.7	$C_{61}H_{96}O_6$+NH_4	C58:11	TAG(18:1/20:5/20:5)
