# Supplementary material for: Immortalized stem cell-derived hepatocyte-like cells: An alternative model for studying dengue pathogenesis and therapy
Source: PLoS Negl Trop Dis. 2020 Nov 20;14(11):e0008835. doi: 10.1371/journal.pntd.0008835 (PMC7717553; doi:10.1371/journal.pntd.0008835)
Supplement: S2 Table — (PDF) [file pntd.0008835.s014.pdf]

S2 Table: CC50 and EC50 values of ivermectin and ribavirin

**Drug**	CC50 values (μM)*			EC50 values (μM)**		
	imHC	Huh-7	HepG2	imHC	Huh-7	HepG2
Ivermectin	9.50 ± 0.86	12.1 ± 0.07	11.25 ± 0.56	5.33 ± 0.82	4.64 ± 0.82	7.97 ± 1.31
Ribavirin	>250	N.D.	N.D.	9.80 ± 0.86	N.D.	N.D.

N.D. – not determined

*Cytotoxicity of different concentrations of ivermectin (1- 50 μM) and ribavirin (3 - 250 μM) was determined on hepatic cell lines following the 48 h of drug treatment. After 48-h incubation, propidium iodide (PI) staining followed by flow cytometry was used to evaluate cell viability. **To determine effective drug concentrations, cells were infected with DENV-2 (16681) at MOI of 0.1 for 2 h. After extensive washing, the cells were cultured in complete media containing different concentrations of drugs in their control vehicle. At 48 h post infection, the cells were harvested and the percentages of DENV-infected cells were evaluated by positive staining of intercellular NS3 expression. Data from triplicate experiments were analyzed to determine the CC50 and EC50 values using GraphPad Prism software.
