# Supplementary material for: Immortalized stem cell-derived hepatocyte-like cells: An alternative model for studying dengue pathogenesis and therapy
Source: PLoS Negl Trop Dis. 2020 Nov 20;14(11):e0008835. doi: 10.1371/journal.pntd.0008835 (PMC7717553; doi:10.1371/journal.pntd.0008835)
Supplement: S3 Table — (PDF) [file pntd.0008835.s015.pdf]

S3 Table: Viability of hepatic cell lines after DENV infection and drug treatment

**Condition**	**Cell viability (% of mock-infected condition)***		
	**imHC**	**Huh-7**	**HepG2**
Medium	90.5 ± 21.4	92.1 ± 17.3	107.5 ± 20.2
Ribavirin	97.8 ± 19.7	78.0 ± 8.1	101.7 ± 12.6
DMSO	99.0 ± 23.4	86.3 ± 5.7	90.4 ± 14.6
Ivermectin	96.4 ± 20.8	78.5 ± 4.5	94.6 ± 22.0

*The three cell lines were cultured in 96-well plates prior to mock infection or infection with DENV-2 (MOI of 0.1) in the plain medium or medium containing DMSO or drugs (5 µM of ivermectin or 10 µM of ribavirin) for 48 h. After 48 h of DENV infection and drug/control treatment, viability of three hepatic cell lines was determined using ATPlite luminescence assay, following the manufacturer's instructions (PerkinElmer Inc.). The cell viability of mock-infected cells was used as a control. The cell viability value from each condition/cell type was calculated as % of cell viability in the mock control condition. Data from triplicate experiments were analyzed to determine the mean ± SD values using GraphPad Prism software.
